# Field enhancement of electronic conductance at ferroelectric domain walls

**DOI:** 10.1038/s41467-017-01334-5

**Published:** 2017-11-06

**Authors:** Rama K. Vasudevan, Ye Cao, Nouamane Laanait, Anton Ievlev, Linglong Li, Jan-Chi Yang, Ying-Hao Chu, Long-Qing Chen, Sergei V. Kalinin, Petro Maksymovych

**Affiliations:** 1 Center for Nanophase Materials Sciences, Oak Ridge National Laboratory, Oak Ridge, TN 37831 USA; 2 Institute for Functional Imaging of Materials, Oak Ridge National Laboratory, Oak Ridge, TN 37831 USA; 3 Multi-disciplinary Materials Research Center, Frontier Institute of Science and Technology, Xi’an Jiaotong University, Xi’an, Shaanxi 710049 China; 4 Department of Physics, National Cheng Kung University, Tainan, 70101 Taiwan; 5 Department of Materials Science and Engineering, National Chiao Tung University, Hsinchu, 30010 Taiwan; 6 Institue of Physics, Academia Sinica, Taipei, 11529 Taiwan; 7 Department of Materials Science and Engineering, Pennsylvania State University, University Park, PA 16802 USA

## Abstract

Ferroelectric domain walls have continued to attract widespread attention due to both the novelty of the phenomena observed and the ability to reliably pattern them in nanoscale dimensions. However, the conductivity mechanisms remain in debate, particularly around nominally uncharged walls. Here, we posit a conduction mechanism relying on field-modification effect from polarization re-orientation and the structure of the reverse-domain nucleus. Through conductive atomic force microscopy measurements on an ultra-thin (001) BiFeO_3_ thin film, in combination with phase-field simulations, we show that the field-induced twisted domain nucleus formed at domain walls results in local-field enhancement around the region of the atomic force microscope tip. In conjunction with slight barrier lowering, these two effects are sufficient to explain the observed emission current distribution. These results suggest that different electronic properties at domain walls are not necessary to observe localized enhancement in domain wall currents.

## Abstract (web summary)

Understanding the conductivity at the nominally uncharged domain walls in ferroelectrics is still far from complete. Here the authors report an enhanced conduction at domain walls in an ultra-thin (001) BiFeO_3_ film resulting from the formation of a field-induced meta-stable twisted domain nucleus.

## Introduction

Localized metal–insulator transitions and other spatial modulations of electronic conductivity in complex oxides have been studied for more than two decades, with many celebrated examples including observation of a percolative transition in doped manganites^1^, formation of a two dimensional electron gas at the interface between two insulators^2, 3^, and conductivity at phase boundaries^4^ and domain walls in both proper^5^ and improper ferroelectrics^6, 7^. The case of enhanced conductivity at the nominally uncharged ferroelectric domain walls was a particularly surprising finding^8^, as good ferroelectrics are generally conceived of as insulators, although in reality they are commonly semiconductors with wide bandgaps (>2.5eV^9^). Perhaps even more surprisingly, the ferroelectric domain wall conductivity (and in some cases, photoconductivity^10–12^) has now been found in a host of systems including BiFeO_3_^8^, doped and mixed-phase BiFeO_3_^4, 12–14^, LiNbO_3_^15^, LiTaO_3_^16^, BaTiO_3_^17^, and PbZr_*x*_Ti_1-*x*_O_3_^18, 19^_._ It is important, in reviewing the literature on the topic, to isolate two categories of experiments: those involving strongly charged domain walls, which are typically unfavored (due to high-electrostatic energy penalty, which will be proportional to energy required to generate free carriers to screen the resulting charge, i.e., the bandgap in the absence of mobile screening charges), and those of uncharged or nominally uncharged domain walls. Given the difficulty in producing charged domain walls in standard ferroelectrics, it is not surprising that their reports are fewer in the literature^14, 17, 20^. The classical explanation for their metallic conductance is the accumulation of carriers (electrons, holes) at the charged domain walls to screen the polarization charge at head-to-head or tail-to-tail walls, resulting in the formation of a quasi 2D electron gas (although in reality the sheet has finite thickness on the order of the screening length)^21^. Similar phenomena were also found to exist in nanodomains formed by an atomic force microscope tip^16, 18^. In contrast, the explanations for domain wall conduction in nominally uncharged, or weakly charged domain walls appear more scattered. Various reports have suggested that the domain wall forms conducting paths through the film^22^ (similar to conduction channels in resistive switching memories), or that the reason for the conduction is the lowered bandgap^23^, with ionization of oxygen vacancies or vacancy clusters^8, 24^, providing the free carriers for conduction localized to the domain wall. Hysteretic behavior has also been found at these domain walls^25^, and transient persistent conduction was reported by Stolichnov et al.^26^, where conductivity was isolated not to existing domain wall positions, but to their prior positions after their motion (induced by the applied field from the atomic force microscope tip). Clearly, a mechanism that posits intrinsically higher conduction at walls cannot be reconciled with a persistent conductivity that is observed and relaxes at previous wall positions. It has however been acknowledged that defect states play an important role in modulating the conductivity, typically oxygen vacancies that are ubiquitous in perovskites, and the conductivity of both domains^27^ and domain walls^24, 28, 29^ appears to be heavily influenced by their concentration.

Given that the studies on wall conduction are often conducted using conductive atomic force microscope (c-AFM) technique, progress in reconciling experiments to possible mechanisms requires a strong appreciation of the role of the junction between the metal-coated AFM tip and the domain wall^7^. An understanding of the inhomogeneity of the electric field generated by the biased tip is also needed, as the field will differ for the same applied potential based on the surrounding polarization profile. In parallel, there has been renewed interest in exotic domain topologies, spurred by the discovery of closure states^30–32^, quadrupole chains^33^, and continuous polarization rotations^34, 35^ in ferroelectric thin films and, very recently, experimental demonstration of vortex-anti-vortex arrays in an oxide superlattice heterostructure^36^. Key to these novel states is the existence of non-zero curl of polarization. Yet the ability to twist existing domain walls (themselves topological defects, according to Mermin’s definition^37^) by the applied electric field and generate ∇ × **P**≠0 has not been heavily explored experimentally. Although such twisted structures are meta-stable, this is precisely the situation incurred during c-AFM studies on ferroelectrics^38^, and warrants investigation. Additionally, distinguishing between the conduction at the domain walls and the bulk of the film would benefit from ultra-thin samples (<~10 nm) (as current will invariably spread into the bulk^7^) while conductivity has only been reported in thicker films (>~30 nm).

In this study, we perform c-AFM measurements in ultra-high vacuum (UHV) conditions on ultra-thin (~10 nm) (001)_pc_-oriented BiFeO_3_ thin films (note, pc refers to pseudocubic) grown on a (110) dysprosium scandanate substrate, and measure conductivity at pre-existing domain walls across the surface of the film. Analysis of high-resolution current–voltage (*I*–*V*) spectroscopy experiments shows that the Schottky barrier is modified near the domain walls at the surface, changing on average by ~20 meV, but the conduction mechanism between the domains and domain walls does not change. Importantly, phase-field modeling reveals the formation of a complex polarization nucleus in conjunction with domain wall twist. This structure enhances the electric field locally by between 1- and 50% at the tip apex, depending on the applied potential, with the nucleation of the reverse domain substantially enhanced near the domain wall. These results show an unexpected merging of domain wall conduction with field-induced topological defects, wherein the field introduces a domain topology where ∇ × **P**≠0 for the twisted domain wall, and which increases the local electric field, allowing for a measurement of the intrinsic conductivity of the underlying ferroelectric. Our findings support a mechanism whereby domain walls need not host intrinsic conductivity, but rather act as nucleation centers for a twisted structure, which serves as field-amplifying confined sites that gate the ferroelectric at nanoscale dimensions. This picture of induced conductivity by geometric field confinement is general and can be widely applicable not only to domain walls, but to any configuration of order parameter topological defects where twisted structures can favorably nucleate.

## Results

### Domain imaging of ultra-thin BiFeO_3_

**Fig. 1:**
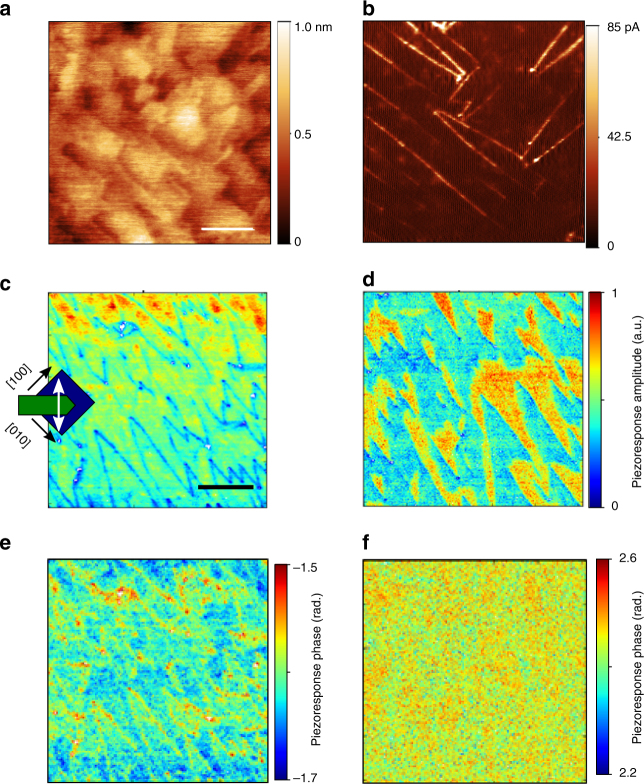
Atomic force microscopy (AFM) measurements of ultra-thin BFO film. **a** AFM Topography and **b** c-AFM of the BFO sample in UHV conditions, with *V*_sam_ = + 1.2 V. Scale bar in **a**, 100 nm. BE-PFM experiments were performed on the same film in ambient conditions (different location to (**a**, **b**)), with the results shown for vertical BE-PFM amplitude in **c** and lateral BE-PFM amplitude in **d** for the same region. The respective phase maps are shown in **e**,**f**. Scale bar in **c**, 500 nm. Orientation of the sample and cantilever for the BE-PFM scan is shown in **c**

The topography of the (001) ~10 nm-thick BiFeO_3_ (BFO) thin film is shown in Fig. 1a, and indicates a smooth surface with low roughness (RMS~200 pm) (Method). The piezoelectric response is weak, and out-of-plane piezoresponse force microscopy (PFM) images show only weak features, while the phase appears mostly uniform, suggesting a uniform out-of-plane polarization component (not shown). Band-excitation PFM imaging in ambient conditions, however, reveals the presence of a mixed stripe domain structure, consisting of predominantly 71° and some 109° domain walls as is the norm for (001) BiFeO_3_ samples on (110)_pc_ DyScO_3_ (Fig. 1c, d)^39^. It should be noted that the phase images from the PFM are inconclusive as the signal was extremely weak given the ultra-thin nature of the films; nevertheless the absence of substantial contrast in the Vertical PFM phase signal means one can conclude that the majority of the walls are 71°, a conclusion which is also supported by x-ray diffraction microscopy^40^ data (Supplementary Fig. 1). The conductive AFM image in UHV, with the tip grounded and the sample biased at *V* = + 1.2 V, is shown in Fig. 1b. The domain walls in this ultra-thin BFO sample display conduction, and as such is the first report of this phenomena in ultra-thin ferroelectric films (though not entirely surprising). Conductivity at junctions between domain walls appears enhanced at some locations, although hot spots are also evidenced along segments of the domain walls, which has been reported earlier for thicker BFO films^25^.

**Fig. 2:**
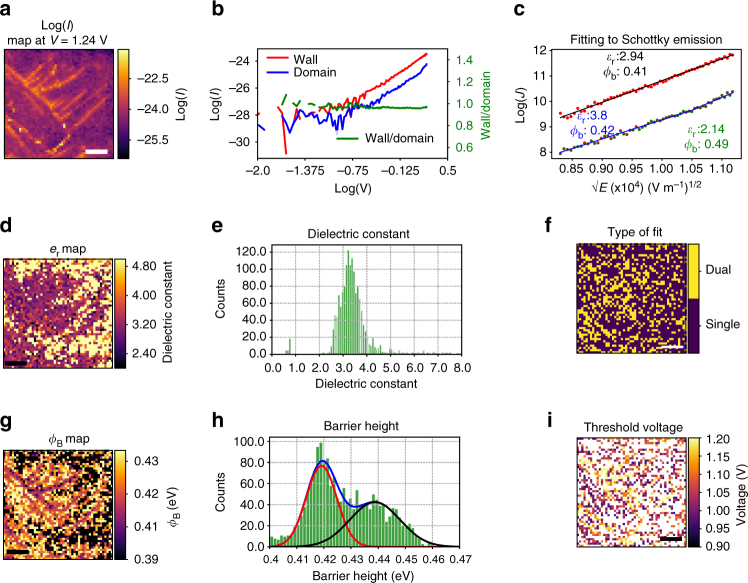
*I*–*V* spectroscopy on ultrathin BFO in UHV conditions. **a** log(*I*) map at *V* = 1.24 V from the *I*–*V* spectroscopic experiment (the voltage is applied to the sample). Two representative points from the domain and domain wall are shown in **b** in a log–log scale. The wall current/domain current is plotted on the right-side *y*-axis, and shows near constant values indicating same mechanism. **c** Two example fits from the data, plotted in the Schottky representation. For one of the points, the fit is best described by a single line fit (black line). The extracted parameters for the dielectric constant and the barrier height are indicated alongside the data. For other points, a two-segment fit is more appropriate (blue line). Barrier height and dielectric constant for both segments are indicated (blue: first segment, green: second segment). **d**, **e** Spatial map and histograms of the dielectric constant, and **f**, **g** spatial map and histograms for the barrier height, computed as discussed in the text. **h** Bayesian information criterion allows assessment of which model (single or dual linear segments) is more feasible; this is mapped spatially. **i** For those points which can be best fit by two linear segments, the threshold voltage (from the first segment to the second segment) is plotted. Scale bars in **a**, **d**, **f**, **g**, **i** are 100 nm

We explore the underlying conduction mechanism further, by performing *I*–*V* spectroscopy in a 50 × 50 grid over a 500 nm × 500 nm area of the BFO film. The log of the measured current at *V*_sam = _1.24 V is shown in Fig. 2a. Similar to the c-AFM measurements, the conductivity enhancement in the vicinity of the domain walls is readily observed. The data was initially pre-processed through use of principal component analysis, for denoising (Supplementary Note 2 and Supplementary Fig. 2). Two selected representative curves from the domain and the wall regions are shown in Fig. 2b, plotted on a log–log scale. The *I*–*V* curves on the domain face and the domain wall itself appear to be very similar. We further plotted the ratio of wall and domain currents, shown as a solid green line. The remarkable stability of this curve through the measured voltage range suggests that the mechanism driving the conduction at the domain walls and the domains themselves is the same, as suggested by some of the earlier works^22, 41^. To identify the conduction mechanism, we began by attempting to fit the *I*–*V* curves to candidate conduction models. Of all the mechanisms investigated (Schottky emission, Poole–Frenkel, space-charge limited conduction (SCLC), Fowler–Nordheim tunneling, and variable range hopping), Schottky emission returned the most reasonable results, as reported in ref. ^22^. Specifically, we fit to

1 $$J \propto {T^2}\exp \left[ { - \frac{{q\left( {{\Phi _{\rm{B}}} - \sqrt {\frac{{qV}}{{4\pi {\varepsilon _{\rm{r}}}{\varepsilon _0}d}}} } \right)}}{{{k_{\rm{B}}}T}}} \right]\,,$$

where *J* is the current density, *T* is the temperature (298 K here), ${\Phi _{\rm{B}}}$ is the barrier height, *V* is the applied voltage, *ε*_r_ is the high-frequency dielectric constant, *ε*_0_ is the permittivity of vacuum, *d* is the film thickness (=10 nm), and *k*_B_ is Boltzmann’s constant. The Schottky emission mechanism dictates that the log(*J*) vs. $\sqrt V$ will be linear, in agreement with the *I*–*V* data, as shown in Fig. 2c (more examples are shown in Supplementary Fig. 4). We note here that the SCLC behavior also returned reasonable values (slopes varied between 3 and 4, which are higher than that reported in ref. ^22^ suggesting a difference in the trap distributions between our work and that by Noheda and Farokhipoor), but the residuals from fitting were slightly larger than for the Schottky emission fit. We therefore utilized Schottky equation as opposed to SCLC (see extended discussion in Supplementary Note 3). An example of a Schottky fit for a single point *I*–*V* curve taken from the spectroscopic measurement in Fig. 2a is shown as a solid black line in Fig. 2c. The barrier height and the (high frequency) dielectric constant appear to illustrate reasonable values (the dielectric permittivity for BFO at high frequency is ~6^42^). However, upon fitting it was evident that many *I*–*V* curves captured during the spectroscopic experiment do not conform to the single linear equation. Rather, they appear better suited to a two-linear segment model (i.e., linear up to a particular threshold voltage, and then linear again after this voltage but with different slope). An example of such data is shown in Fig. 2c with a two-linear segment fit in blue. The dielectric constant for the first segment is reasonable, but appears slightly less reasonable for second segment fit. It should be noted that the fitting range for the *I*–*V* curves was 0.68 < *V* < 1.25 V, and robust fitting was used to exclude the effects of outliers. To explore this more systematically, we employed the Bayesian information criterion approach (BIC^43^, see also Supplementary Note 3) in determining whether the linear or two-linear segment model is more suitable for the curve fitting at each point. Where the single linear fit was more suitable, we employed values for the barrier height and the dielectric constant from those fits; where two linear segments were the more likely model (according to BIC), we used the values of the first segment of the fit. Maps and histograms of the dielectric constant and the barrier height are shown in d–h, and reveal that the dielectric permittivity is mostly uniform (though a substantial proportion of pixels show higher values than expected), whereas the barrier height is slightly lowered at the domain walls. The magnitude of this shift in the barrier height is confirmed via Gaussian deconvolution of the peaks in the histogram of the barrier height, in Fig. 2h. The difference appears to be about ~20 meV between the domain walls and the domains. While the change in barrier height is quite small, it is still on the order of ~5% of the original barrier height. The values for the barrier height are noticeably smaller than expected, for a Pt/BFO interface^44^. This is a feature that has been observed previously, and has been addressed by various authors^45, 46^ through modification of the barrier heights measured through addition of a space-charge term.

Shown in Fig. 2f is a map of the individual locations (pixels) where each model (single or two linear-segment fit) is preferred. Many of the points where a transition exists occur at the domain walls, though points within domains which display this behavior are also found. The threshold voltage for the cross-over is plotted in Fig. 2i, and appears to be quite high—typically over 1 V, and is especially high at the domain walls. Due to the high threshold voltage, the smaller number of data points (i.e., the voltage steps for the second segment for these *I*–*V* curves) precludes further detailed investigations of the conduction mechanism. Nonetheless, the results in Fig. 2 suggest that there exists some threshold voltage for a change in the mechanism, and further, that the mechanism for the conduction is the same for the domains as for the walls.

### Phase-field modeling of the polarization nucleus

To investigate the origin of the observed changes in Schottky emission at a domain wall, we turned to phase-field modeling. The latter has been used extensively over the past decade in modeling the elastic and electric configurations of domain structures in ferroelectric thin films^47, 48^. A central aspect of c-AFM measurements reported here and in previous investigations of domain wall conductivity are the very large local electric fields generated by the biased AFM tip, which is expected to cause substantial polarization reconstruction and consequently modify the local electric field distribution. One of the key assumptions in such investigations is that given a fixed applied potential to the AFM tip (or sample), the field distribution is identical across the sample, so that the conductivity values obtained from c-AFM are directly comparable from site to site. This assumption does not hold generally in ferroelectrics, given that the applied field couples to the initial polarization configuration which is variable across the surface, especially across domain walls. We directly quantified the degree to which this effect has an impact on the measured conductivity by phase-field modeling (details of modeling are given in Supplementary Note 4).

**Fig. 3:**
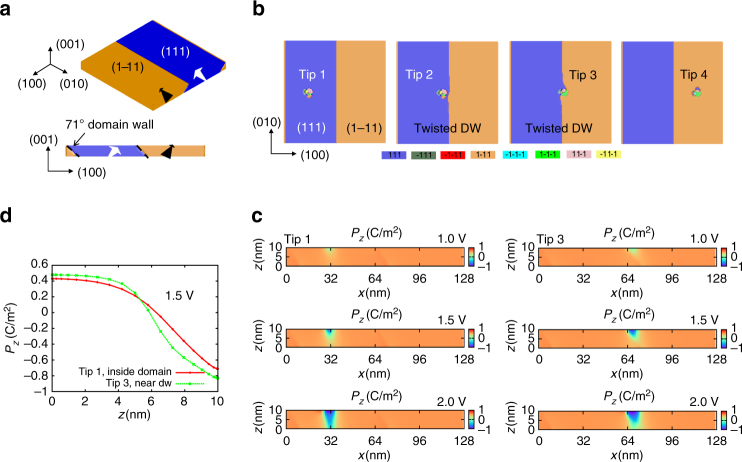
Phase-field modeling of 71° domain walls in BFO and formation of a twisted polarization nucleus. **a** Model setup indicating orientations of the domains. **b** *x*–*y* cross-sections at *V*_tip_ = 1.5 V for the tip placed at four distinct locations indicating the polarization orientations. Color legend indicates polarization orientations for each domain. **c** *x*–*z* cross-sections showing polarization distributions for the *z*-component of polarization (*P*_*z*_) for the tip in locations 1 and 3, as a function of voltage applied to the tip. The *z*-component of polarization profile taken from the tip to the bottom surface is plotted in **d** for two of the tip positions

To probe the field enhancement at domain walls under the concentrated electric field of an AFM tip, we simulated a 10 nm thick film consisting of (111) and (1–11) domains separated by 71° domain walls as shown in Fig. 3a. The tip (*γ* = 5 nm, see Supplementary Note 4) is then placed at points in both domains as well as on both sides of the domain wall before the polarization structure is evolved under the applied potential in each case. The domain structure in the (*x*–*y*) planes is shown in Fig. 3b for four different tip locations (*V*_tip_ = 1.5 V), while the (*x*–*z*) cross-sections are shown in Fig. 3c. These simulations reveal in every case the field-induced formation of a complicated nucleus, as the local polarization begins to align to both the in-plane and out-of-plane components of the applied electric field. In the ideal case, the result will be a center domain (radially symmetric, with soft restriction of orientation of **P** to crystallographically allowed orientations)^49^. However, when the tip is near the domain wall, there appears a twisted wall structure, where in one case the wall is bowed towards the nucleus (tip 2 in Fig. 3b) and in the other case, the wall is twisted clockwise to wrap around the nucleus (tip 3 in Fig. 3b). Furthermore, the reverse-domain nucleus extends deeper when the tip is near the domain wall, than when the tip is within the domain, as seen in the polarization profile in Fig. 3d.

**Fig. 4:**
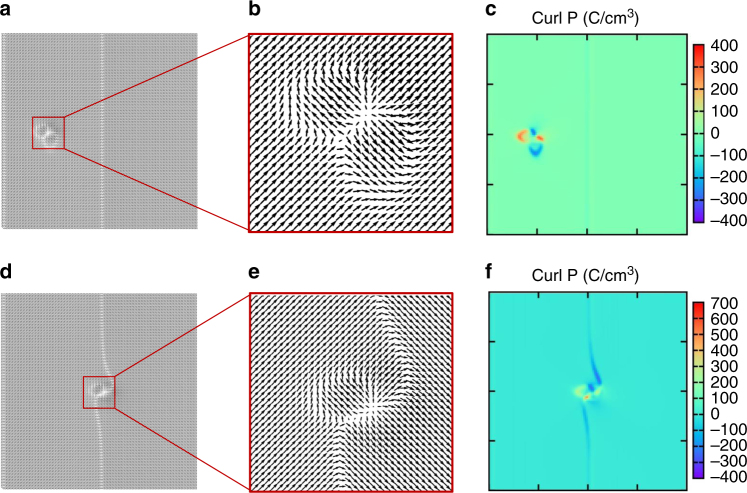
Domain nucleus and polarization-twist structure with non-zero curl. Phase-field simulated polarization vector plots, for the (*x*–*y*) plane at the surface are shown in **a**, **b**, **d**, **e** for the case when a voltage of *V* = 1.5 V is applied to the tip, for two different tip locations. The curl of polarization $\left( {\nabla \times {\bf{P}} = \left( {\frac{{\partial {P_y}}}{{\partial x}} - \frac{{\partial {P_x}}}{{\partial y}}} \right)\hat {\bf{k}}} \right)$ is also plotted **c**, **f** for each case. **a**–**c** Corresponds to the tip located within the domain (tip 1 in Fig. 3), whereas **d**–**f** corresponds to the situation where the tip is adjacent to the domain wall (tip 3 in Fig. 3)

To further identify the nature of the twisted polarization nucleus, we plot the polarization vector fields for the biased tip (*V*_tip_ = 1.5 V) in locations 1 and 3, in Fig. 4a, d, for the surface *x*–*y* plane. The curl of the polarization, i.e. ${\nabla \times {\bf{P}} = \left( {\frac{{\partial {P_y}}}{{\partial x}} - \frac{{\partial {P_x}}}{{\partial y}}} \right)\hat {\bf{k}}}$ is also plotted in Fig. 4c, f for each tip location. Interestingly, non-zero curl exits for both situations, but the curl is substantially higher for the case where the tip is next to the domain wall (and the wall twists). Moreover, the volume of film in which the polarization is affected is more substantial for the tip in location 3 than for the first case. This is reflected in the polarization maps of both surface and bulk, and is due to the reason that twisting of the pre-existing domain wall incurs substantially less energy penalty than creating a new domain to align with the in-plane component of the field. This can be contrasted with the case in tip 1, where the simulation shows a high-energy interface with the surrounding matrix, and therefore a smaller area of polarization reversal. We note that the non-zero curl is a situation that is classically forbidden by Maxwell’s laws for electrostatics for an applied electric field; however, they can exist for the polarization field, and further such structures can be meta-stable arising from the dynamics of polarization reversal. For investigating the polarization structure in the film bulk, a line profile of the *z*-component of polarization through the *z*-direction from the tip apex to the bottom of the film is plotted in Fig. 3d. From these plots, there is substantial asymmetry in the results, because of the different polarization pattern for the tip placed at position 1 and position 3. Here it appears that the polarization reversal proceeds to greater depth when the tip is placed in location 3, and indicative of a greater divergence of polarization (slightly away from the surface) for the tip in this location (see plot in Supplementary Fig. 7).

**Fig. 5:**
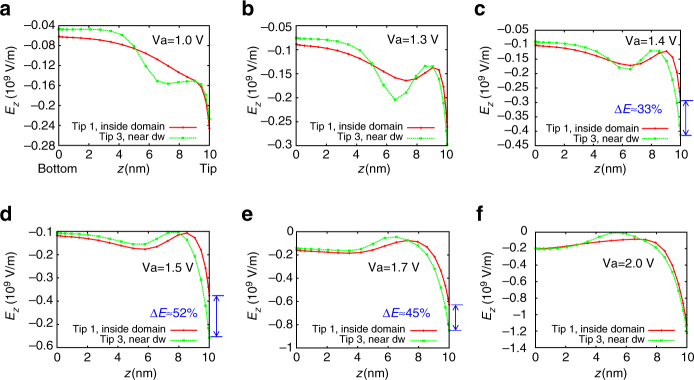
Phase-field modeling the enhancement of field due to polarization-twist structure, as a function of potential applied to the tip. Results are plotted in **a**–**f** for progressively larger tip voltages. As the tip voltage is increased, the electric field profile within the film changes in response to the polarization redistribution. The degree of field enhancement is calculated by determining the electric field at the apex for the case where the tip is near the domain wall (tip 3), as compared to when it is contained within the domain (tip 1)

To explore the modulation of the local electric field by domain wall twisting, we plotted the (calculated) *z*-component of the electric field, in Fig. 5, for tip locations in the center of the domain (tip 1) and the tip near the domain wall (tip 3), for different values of applied tip potential. We found that the wall-twist structure and its interaction with the nucleus alters the field profile substantially, leading to enhancement of the field magnitude at the surface when the tip is located at the domain wall, compared to when it is within a domain. The field profile as a function of depth shows a local maximum that is deeper within the film for the tip near the domain wall (and arises as a result of the polarization charge at the interface between the reverse domain and the surrounding matrix). This results in a field enhancement at the surface of the domain wall (*z* = 10 nm), which is small at 1.0 V, but which grows with increasing voltage to ~50% for an applied tip potential of 1.5 V. This result shows that application of an electric potential to the tip will cause local-field enhancement, due to the polarization nucleus structure formed. In fact, such enhancement will even exist for the purely linear dielectric case;^50^ however, the enhancement is much greater for the domain wall-twist structure, as a result of a much more labile domain wall and lowered nucleation barriers for the reverse domain.

**Fig. 6:**
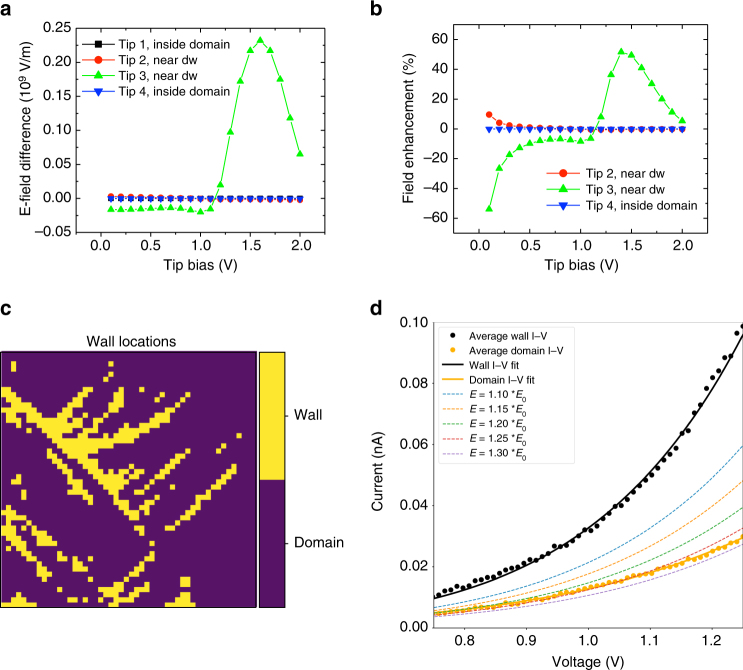
Electric-field enhancement due to the polarization-twist structure as mechanism of wall conduction. **a** Electric field difference at the apex, from phase-field modeling for the four different tip positions (from Fig. 3). Difference is taken with respect to tip 1. **b** Field enhancement from the tip placed at locations 2–4, as a function of tip bias. **c** Map of domain wall and domain locations identified from the spectroscopic experiment in Fig. 2. **d** Mean *I*–*V* curves taken on the domain walls (yellow pixels in **c**) and on the domains (purple pixels in **c**), plotted as black and orange circular markers, respectively. Fits to Schottky emission equation are shown as black and orange curves, respectively. Further dashed lines are plotted assuming field intensification at the domain wall by values ranging from 10 to 30%. The normalization serves to reduce the current observed at the wall, and assuming 25% field enhancement, the observed current at the wall is similar to that obtained within the domain

We further quantified this behavior for different tip positions, plotting the differences in the electric field at the tip–film interface when compared with the tip placed within the domain in Fig. 6a. The result indicates a large difference when the tip is placed at the domain wall, with nonlinear field enhancement, which reduces after ~*V*_tip_ = 1.5 V. The fractional degree of field enhancement is plotted in Fig. 6b, and again highlights both the nonlinear nature of the enhancement, as well as the large asymmetry between the tip at positions 2 and 3, both of which are near the domain wall. This result also suggests that the wall conduction should be asymmetric, which is partially supported by observations (Supplementary Fig. 8). To observe the effect of the enhanced local fields on the conduction that would be observed in measurements, we plot the average *I*–*V* from the domain wall regions in Fig. 6d, and compare it to the domain face regions (black and orange markers). The respective Schottky emission fits (per equation (1)) are shown as solid black and orange lines. In addition, we plot the expected domain wall current in the case that the field is locally enhanced by amounts ranging from 10 to 30%, i.e., we renormalize the wall conduction based on the expected field enhancement, for comparison with the domain conduction. These results show that a field enhancement of ~25% at the domain wall can result in current values that adequately match the domain face conduction. However, the slopes are still distinct, which can be rationalized by a combination of non-linear field enhancement (the enhancement in the electric field is not a linear function of the applied potential, as explained above), change in barrier height (which would be possible from the re-orientation of the polarization and associated barrier lowering^26^), and potentially, the change in the dielectric permittivity around domain walls. Such a scenario can explain most of the conductivity ‘enhancement’ seen around the domain walls. Moreover, the field enhancement appears to occur at a threshold voltage. Practically, this is likely to vary substantially in the real experiment due to local geometry of the domain walls (which are more complex than the simple structures modeled), distribution of pinning sites, presence of chemical or structural inhomogeneities, etc. Nonetheless, we do observe that at many points, there appears to be a cross-over in the slope, as evidenced in Fig. 2, providing some support for this proposed mechanism.

## Discussion

We note that repeated scans by the AFM tip, by disabling the slow-scan axis, appears to increase the domain wall conductivity (Supplementary Fig. 9). We reason these effects are likely due to a combination of redistribution of mobile charges affecting the tip–surface junction properties, and stabilization of the complex twist structure. The work by Stolichnov et al.^26^ showed persistent conductivity after domain walls had moved from initial positions, and other related time dynamics of wall conductivity^25^ have been reported previously. Our report here is consistent with those observations, namely, the mechanism suggested in that study was the change in barrier height due to the unscreened polarization in the freshly switched area. However, in our case we do not need to invoke defects to explain the apparent domain wall conductance. Further, the key differences in our study are that we posit that the domain wall becomes conducting through essentially field-induced excitation of the polarization order parameter, and therefore its conductivity is a transient effect. This is in contrast to models with strong carrier accumulation and metallicity that imply the domain walls to remain conducting at zero field.

We also note that when the domain wall is reconstructed through the formation of the twist structures, one is no longer actually measuring the DC conductance of the virgin domain wall but rather that of the field-induced topological defect. In fact, the polarization nucleus structure would be formed in both the domains and the domain walls, but when the tip is placed near the domain wall, the twisting of the domain wall at low potential allows much more substantial polarization re-orientation to occur, greatly modifying the field and resulting in non-linear enhancement of the field at the tip apex across the range of potentials studied in experiment. We may conclude that the observed current enhancement at domain walls need not be an intrinsic property of the domain wall itself, but can be from the topological structure that arises when large fields are applied via the AFM tip. This argument would certainly suggest that the conduction mechanism of wall conduction is the same as that of the surrounding domains, which is mostly observed in the case of the nominally uncharged domain walls^22, 41^.

This mechanism to explain domain wall conductivity relies on no electronic property changes at domain walls, and does not invoke oxygen vacancies. Naturally, the concentration of oxygen vacancies and cation vacancies^29^ can play a substantial role in the material conductivity, and can be preferentially accumulated at domain walls (e.g., due to either electrostatic or strain-gradient effects^5^), but generally for uncharged walls the mechanism of domain conduction remains the same as for the domain walls^22, 41^, and the enhancement is not in general very large. A similar charged domain twist structure was invoked previously, for the conductivity observed at ferroelectric vortex cores^51^. Here we build on the concept, and suggest it can be generalized to explain the observed conductivity of both domain walls and domain junctions. The question also arises of whether such wall twists can be observed experimentally. We note much experimental evidence exists to show that such wall complex structures are indeed possible in both rhombohedral^49, 52, 53^ and tetragonal^54–56^ systems at high fields, some of which have also been modeled by phase-field simulations^49, 57, 58^. Bowing of the domain wall has also been directly imaged^59^, and modeled analytically^60^. In addition, there is a wealth of evidence in the last decade, primarily by scanning transmission electron microscopy, for polarization topologies with non-zero curl^30, 34, 36, 61^. Based on this data, we suggest that extrapolation of the phase-field simulations to lower fields is justified.

We finally note that given that the conduction is interface limited, this limits our understanding of the bulk conduction mechanism^62^. It can be said that there should be effects on the emitted electrons due to interaction with trap states, and that bulk mobility is also a parameter in the modified form of the Schottky equation (valid for cases where the mean free path of the electron is smaller than the thickness of the dielectric). Recent work has suggested that the intrinsic AC conductance of domain walls is indeed different^63, 64^, as explored through scanning microwave impedance microscopy, where it was posited that due to domain wall roughness induced by disorder, local perturbations of the wall can accumulate charge even in nominally uncharged walls^63^.

In summary, we propose a mechanism for apparent conductance of ferroelectric domain walls, relying solely on the field-induced changes to ferroelectric topology. Based on extensive phase-field simulations of polarization changes induced by the AFM tip, we infer local electric field enhancement at the tip apex from a complicated polarization-twist structure with non-zero curl that facilitates interfacial electronic conduction. The mechanism presented here can reconcile some discrepancies in the existing literature, and strongly suggests that the field-induced perturbations to the domain structure should be taken into account in conductive AFM measurements. This mechanism can be generalized to explain observed conduction at domain vertices and other topological defects in ferroelectrics.

## Method

### Film growth and characterization

The ~10 nm BiFeO_3_ thin films were deposited on etched (110) DyScO_3_ substrates via pulsed laser deposition with a KrF excimer laser (248 nm) striking a stoichiometric target at 700 °C in an oxygen pressure of 100 mTorr and cooled in 1 atm of oxygen. The BiFeO_3_ thin films have high crystal quality (rocking curve FWHM ~120 arcsec, DyScO_3_ FWHM ~12 arcsec) and are coherently strained to the substrate (Supplementary Note 1). The Band-Excitation PFM measurements were performed in ambient environment in an Asylum Research AFM, with National Instruments PXi-based hardware and in-house scripts written in Matlab and Labview.

### AFM measurements

UHV PFM and c-AFM measurements were performed at room temperature in an Omicron VT AFM/STM system. In all cases, the AFM cantilever used were Budget Sensors ElectriMulti75-G Cr/Pt coated tips with a nominal force constant of 3 N/m. For UHV experiments, the tip was grounded and the sample was biased for all biasing measurements.

### Data analysis

All numerical analysis was carried out in Python v 2.7 (Anaconda package). Before fitting of the *I*–*V* data, the data was de-noised using principal component analysis, which was successful in eliminating most of the oscillatory noise present in the signal (Supplementary Note 2). Note that points where the fit was poor, as determined by setting a threshold on the residuals, were excluded from the maps and subsequent histogram.

### Data availability

All data used in this manuscript is available from the authors on request.

## Electronic supplementary material

Supplementary Information

Peer Review File
