# Supplementary material for: Field enhancement of electronic conductance at ferroelectric domain walls
Source: Nat Commun. 2017 Nov 6;8:1318. doi: 10.1038/s41467-017-01334-5 (PMC5673066; doi:10.1038/s41467-017-01334-5)
Supplement: Supplementary file 1 — Supplementary Information [file 41467_2017_1334_MOESM1_ESM.pdf]

PEER REVIEW FILE

Reviewers' comments:

Reviewer #1 (Remarks to the Author):

The authors investigate the conductivity of twisted domain wall in an ultra-thin (001) $BiFeO_3$ thin film through ultra-high vacuum conductive atomic force microscopy (c-AFM) measurements. It is found that the domain walls in the ultrathin BFO sample display conduction and the conductivity at junctions between domain walls is enhanced. To identify the conduction mechanism, Schottky emission is found to return a reasonable fitting. Furthermore, the phase field model is employed to simulate the twisted domain structures at the domain walls and in the domains. The localized electric field induced by AFM tip results in a complex polarization twist structure with non-zero curl arising at domain walls. The authors claim a new conduction mechanism relying on field-modification effect from polarization dynamics and the structure of the reverse-domain nucleus with non-zero polarization curl. The observed results may be correct. However, there are a few drawbacks or unclear points the authors should address.
1. In this work, the results of phase field modeling have less correlation with the experimental observations. Phase field modeling of Fig.4, Fig.5 (a)-(b), and Fig. 6 only give the static polarization or electric field distributions under different voltages and loading locations, which are nothing to do with the conductivity due to the absence of the free charges density in the phase field model. To explain the conductivity effect, the phase-field simulation must incorporate coupling between ferroelectric and semiconductor properties, i.e., the Gauss's law of phase field model should include a nonzero concentration of free charges density [Nature Communications|4:1808|(2013); Physics Letters A 377 (2013) 1643–1648].
2. The current-voltage model based on Schottky barrier has already been employed to describe the conductivity behavior of the charged domain walls of single domain ferroelectric crystal by T.Sluka et al. [Nature Communications|4:1808|(2013)]. In fact, the twisted domain walls obtained from the phase field simulation are also somewhat charged domain walls (the divergence of polarization is nonzero). Therefore, their conductivity is intrinsically the same as those of charged domain walls or the vortex core observed in the literature previously, in which the polarization induced charges at the domain walls or vortex core bending the band structure and thus makes the materials conductive.
3. The authors claim a new conduction mechanism relying on field-modification effect from polarization dynamics. I did not see any polarization dynamics in the manuscript. There are no results on the temporal evolution of polarization. Fig.6 only gives the domain patterns under different voltages and the evolution time is absent. Are these domain structures final stable states in the simulations? If yes, where is the dynamics of polarization?

Reviewer #2 (Remarks to the Author):

The submitted paper presents new and very important results and deserves to be published. Conductivity of domain walls in ferroelectrics has attracted significant interest because of its importance for the understanding of the properties of ferroelectric thin films and due to the possibility of the development of electronic devices utilising conductivity of domain walls. A number of experimental and theoretical studies of domain wall conductivity have been performed, however, the nature of domain wall conductivity has not been understood completely . The submitted paper adds important data to the study of domain wall conductivity. Especially interesting are the results of the phase field modeling that indicate that there may exist a new mechanism for the domain wall effect on the conductivity of thin ferroelectric films. I would like to suggest only a few of minor corrections.

The authors should present previously obtained results more clearly. In particular, the paper by S Farokhipoor and B Noheda [Ref. 21 in the submitted paper] presents data on the conductivity of 40 nm thick BFO films and demonstrates that at a sufficiently high voltage both the conductivity of domains and the conductivity of domain walls are controlled by Schottky emission, while at a low (below 3 – 3.5 V) voltage, conductivity is limited by the space charge. The authors of the submitted paper demonstrate that conductivity in 10 nm BFO films is controlled by Schottky emission, however, they have not observed the low voltage space charge limited regime. It is important to note that in the submitted paper, the I(V) curve fitting has been performed for the voltage range $1.0<V<1.25$, which corresponds to high voltage conditions reported in Ref. 21 when the difference in the film thickness is taken into account. A significant difference between the results presented in Ref. 21 and in the submitted paper lies in the estimated effect of the domain wall on the barrier height for Schottky emission which is much stronger according to Ref. 21 than according to the submitted paper. At least a brief discussion of the similarities in and differences between the results (and possible reasons for them) should be presented.

The authors write, "Strikingly, the domain walls in this ultra-thin BFO sample display conduction, a result which has not been reported in ultra-thin ferroelectric films to date." The question is whether such results have not been reported because no studies were performed on ultra-thin BFO films, or whether the studies were conducted but did not demonstrate any conductivity enhancement by domain walls. If the former is the case, then the conductivity effect found in the submitted paper should not be surprising, given numerous data on the domain wall effect on conductivity in thicker films and even in bulk material. If the authors of the submitted paper assume that 10 nm films should be significantly different from 30 nm films in that respect, then the reasons for such an assumption should be provided.

The authors stress several times that the discovered twisted domain wall configuration has a non-zero polarization curl which "is a situation that is classically forbidden by Maxwell’s laws for electrostatics". Maxwell's laws prohibit only a non-zero curl of polarization induced by the applied electric field (as an electrostatic field itself has a zero curl according to Maxwell’s laws). Permanent dipoles produced in a ferroelectric may form a polarization field with a non-zero curl by forming vortex shaped polarization patterns as has already been demonstrated both experimentally and theoretically.

Some of the figures need improvement. In Fig. 2.b, the barrier height and the high-frequency dielectric constant values are presented, however, it is almost impossible to read them. Fig. 6 presents polarization patterns in twisted domain structures, however, they are so small that it is not possible to understand the polarization distribution in them. These problems may be solved if the twisted structures are cropped from a larger domain pattern and presented with a larger magnification. Alternatively, the figures could be moved to the "Supplementary information" section where they could be presented in a larger size.

Reviewer #3 (Remarks to the Author):

A new twist on domain wall conduction by Rama K. Vasudevan et al. reports on the origin of conduction at uncharged domain walls, namely 71o domain walls, in BiFeO3 films. The authors use more sophisticated techniques than previous reports to confirm: a) the mechanisms for conduction of domain and domain walls are the same, that is there is not intrinsic domain wall conduction origin, such as a lower band gap and b) the mechanism is Schottky emission and, thus interface determined. Next to the more robust and statistically more significant data analysis using the PCA noise reduction method, the added value of this paper is to show that conduction of domain walls is also present in films as thin as 10nm.
But the original idea of the paper that justifies its consideration in a high impact journal such as Nature Comm. is the proposal that the increased currents at domain walls are due to locally enhanced electric field, as opposed to locally decreased values of the Schottky barrier due to migration of space charge to the walls, as previously proposed. This argument is supported by phase field simulations showing that polarization curls could form at the domain walls and could eventually amplify the electric fields sufficiently.

Even though I think it is a very interesting idea, certainly worth to be published, I doubt that this journal is the proper place for it, since evidence of those polarization curls is not shown. The authors write that this proposed mechanism does not need the presence of defects, but it is a fact that those defects are present in the films and it has been demonstrated by several authors cited in

the manuscript that changes in the defect concentration (either by oxygen vacancies or by cations) give rise to clear changes in conductivity. So defects need to be taken into account when simulating the electrostatic potential with or without curls. The effect shown in Figure 5 is not convincing enough (differences in slope with the experimental data are assigned to field non-linearity, which will certainly play a role but it is not clear in which direction and how). At the very least the simulations should show the comparative effect of the decrease in Schottky barrier upon defect migration and maybe also polarization re-orientation (effect that one cannot ignore) and the increase of the electric field.

Other points of consideration are:
1) the fit to the Schottky emission I-V curves gives high-frequency dielectric constants of 40-50 which are much larger than the expected values (around 5). In fact the simulations use very similar value for the static dielectric constant.

2) the simulations use a tip of 5 nm (radius?) while the AFM metallic tips are typically 3 times bigger.

3) The samples are 10nm thin while the simulations consider a 32 nm thick film. Why not the same thickness?

4) In general the relevant papers are cited but not always in the proper way. For example: “The case of enhanced conductivity at the nominally uncharged ferroelectric domain walls was a particularly surprising finding, as good ferroelectrics are semiconductors with wide bandgaps (>~2.5eV). “
Reference [11] should be cited after “finding“ as it was the first report on domain wall conductivity.
Another example is also mentioned above: this paper confirms a) the mechanisms for conduction of domain and domain walls are the same and b) the mechanism is Schottky emission and, thus interface determined. Both were reported by the Groningen group against the, then, prevalent models. The way these papers are cited does not properly reflect this fact. E.g. In “To identify the conduction mechanism, we began by attempting to fit the I-V curves to various conduction models. Of all the mechanisms investigated (Schottky emission, Poole-Frenkel, space-charge limited conduction, Fowler-Nordheim (FN) tunneling and variable range hopping), only Schottky emission returned reasonable results.“ one misses “as reported in ref.21”.

Reviewer #4 (Remarks to the Author):

Vasudevan et al. address domain wall conduction in terms of conductive atomic force

microscopy and phase-field simulations. The work contains several new and interesting ideas / results, but additional data is required in order to support the conclusions drawn in the manuscript. After the authors provided such data and improved the discussion, the manuscript might be of interest for the readership of Nature Communications.

General:

1. The manuscript contains some typos, e.g., line 96 should read “domain walls” not “domains”, line 127: “one is missing”, etc.

2. Some references need to be added, e.g.; line 51/52: band gap for “good ferroelectrics”. Line 94/95: “this is precisely the situation incurred during c-AFM...”.

Scientific:

1. The authors write that “only Schottky emission returned reasonable results” (line 143). Fitting of the other relevant models, including a discussion of the fitting constants, should be presented in the supplementary. This way the reader can understand why the model is the only reasonable one, which is quite often not clear in cAFM. I suspect, for instance, that the thermionic emission model by Simmons can describe the data, too. Thus, the presentation of the analysis should be improved.

2. Data for positive and negative bias should be shown in Fig. 2. This could further support the interpretation of the conduction mechanism presented in the paper.

3. Moreover, fits for the domain and the domain wall should be shown in Fig. 2c. It is said that domain and wall exhibit the same conduction mechanism. The domain data, however, shows an intriguing kink that should be addressed.

4. The voltage applied during the cAFM scan is about 5 order of magnitude larger than in previous studies performed on, e.g., LiNbO3 or HoMnO3 bulk materials. Thus, defects at the domain wall and electrical breakdown may start playing a crucial role for the observed conductance. This should be discussed, especially as no intrinsic effect such as the reported twist would be needed to explain the experimentally observed phenomena.

5. Since dynamical phenomena are discussed, time-dependent cAFM data could provide additional insight and support the authors interpretation.

6. Moreover, direction dependent scans (forward and backward) should be presented in Fig. 1. Based on the simulations, I would expect them to look substantially different on 71-degree

domain walls.

7. What is the explanation for the lower dielectric constant observed at the domain walls in Fig. 3a?

8. The simulations in Fig 4 are performed for significantly thicker films than measured by cAFM. Simulation results in the thin film limit should be presented and discussed in order to make the connection to the experiment.

9. I do not fully understand why the twisting caused within a domain (Fig 4c, first panel) does not involve enhanced currents in cAFM? It would be helpful to elaborate this in more detail.

Reviewers' comments:

Reviewer #1 (Remarks to the Author):
The authors investigate the conductivity of twisted domain wall in an ultra-thin (001) BiFeO3 thin film through ultra-high vacuum conductive atomic force microscopy (c-AFM) measurements. It is found that the domain walls in the ultrathin BFO sample display conduction and the conductivity at junctions between domain walls is enhanced. To identify the conduction mechanism, Schottky emission is found to return a reasonable fitting. Furthermore, the phase field model is employed to simulate the twisted domain structures at the domain walls and in the domains. The localized electric field induced by AFM tip results in a complex polarization twist structure with non-zero curl arising at domain walls. The authors claim a new conduction mechanism relying on field-modification effect from polarization dynamics and the structure of the reverse-domain nucleus with non-zero polarization curl. The observed results may be correct. However, there are a few drawbacks or unclear points the authors should address.

We are deeply thankful to all the referees for their insights, comments and criticisms, which have undoubtedly strengthened our manuscript. We have performed a large amount of new analysis and new simulations to better understand and illustrate the mechanism we have proposed, and we believe we have successfully answered the questions posed by all the referees. Both the paper and supplementary files have been modified substantially from our initial submission.

We would like to thank Referee 1 for appraisal of our manuscript and their comments, which we respond to below.

1. In this work, the results of phase field modeling have less correlation with the experimental observations. Phase field modeling of Fig.4, Fig.5 (a)-(b), and Fig. 6 only give the static polarization or electric field distributions under different voltages and loading locations, which are nothing to do with the conductivity due to the absence of the free charges density in the phase field model. To explain the conductivity effect, the phase-field simulation must incorporate coupling between ferroelectric and semiconductor properties, i.e., the Gauss's law of phase field model should include a nonzero concentration of free charges density [Nature Communications|4:1808|(2013); Physics Letters A 377 (2013) 1643–1648].

We consider interface-limited carrier injection as the largest resistance in the equivalent circuit. The Schottky-like potential profile across the interface in our case arises due to a combination of the very small (nanoscale) size of the tip-electrode and due to the screening of the applied potential by polarization distortions. This latter component is primarily responsible for the contrast between domain and domain walls. From our earlier work, the effect of moderate doping on the specific polarization profile appears to be very small (e.g. see Fig. R1), justifying to an extent the dielectric approximation employed here. Finally, given the ultrathin film thickness studied here, we would expect the films to be fully depleted, and well-approximated by the dielectric rather than a semiconductor.

2. The current-voltage model based on Schottky barrier has already been employed to describe the conductivity behavior of the charged domain walls of single domain ferroelectric crystal by

T.Sluka et al. [Nature Communications|4:1808|(2013)]. In fact, the twisted domain walls obtained from the phase field simulation are also somewhat charged domain walls (the divergence of polarization is nonzero). Therefore, their conductivity is intrinsically the same as those of charged domain walls or the vortex core observed in the literature previously, in which the polarization induced charges at the domain walls or vortex core bending the band structure and thus makes the materials conductive.

This is an important point, but the key differences are that we posit that the domain wall becomes conducting through essentially field-induced excitation of the polarization order parameter, and therefore its conductivity is a transient and dynamic effect. This is in contrast to the models with strong carrier accumulation and metallicity that imply the domain walls to remain conducting at zero field. The two mechanisms may well coexist in some materials, but our model will generally precede the formation of the metallic domain wall and should be quite universal among ferroelectrics.

3. The authors claim a new conduction mechanism relying on field-modification effect from polarization dynamics. I did not see any polarization dynamics in the manuscript. There are no results on the temporal evolution of polarization. Fig.6 only gives the domain patterns under different voltages and the evolution time is absent. Are these domain structures final stable states in the simulations? If yes, where is the dynamics of polarization?

We agree with the reviewer that the terminology may be slightly confusing as written. We are dealing with dynamics in the sense that the conducting entities are not a domain or a domain wall in their as-grown state, but rather perturbed polarization configurations, created by the action of electric field on the respective entities. Moreover, the degree of perturbation grows with increasing strength of applied field. However, it is true that here we consider the perturbed domain states to be quasi-static for the given potential. Hence, in this case we deal with the voltage-induced polarization responses, rather than time dynamics.

That said, these configurations associated with mesoscopic changes in polarization distributions are often associated with slow relaxation, giving rise to time dynamics as well (as observed for these domains walls in [Maksymovych et al.[1]])

Reviewer #2 (Remarks to the Author):

The submitted paper presents new and very important results and deserves to be published. Conductivity of domain walls in ferroelectrics has attracted significant interest because of its importance for the understanding of the properties of ferroelectric thin films and due to the possibility of the development of electronic devices utilising conductivity of domain walls. A number of experimental and theoretical studies of domain wall conductivity have been performed, however, the nature of domain wall conductivity has not been understood completely . The submitted paper adds important data to the study of domain wall conductivity. Especially interesting are the results of the phase field modeling that indicate that there may exist a new mechanism for the domain wall effect on the conductivity of thin ferroelectric films. I would like to suggest only a few of minor corrections.

We greatly appreciate the referee's positive comments and appraisal of our manuscript. The comments have greatly improved our manuscript, and we believe we have reasonably answered the questions posed.

The authors should present previously obtained results more clearly. In particular, the paper by S Farokhipoor and B Noheda [Ref. 21 in the submitted paper] presents data on the conductivity of 40 nm thick BFO films and demonstrates that at a sufficiently high voltage both the conductivity of domains and the conductivity of domain walls are controlled by Schottky emission, while at a low (below 3 – 3.5 V) voltage, conductivity is limited by the space charge. The authors of the submitted paper demonstrate that conductivity in 10 nm BFO films is controlled by Schottky emission, however, they have not observed the low voltage space charge limited regime. It is important to note that in the submitted paper, the I(V) curve fitting has been performed for the voltage range $1.0<V<1.25$, which corresponds to high voltage conditions reported in Ref. 21 when the difference in the film thickness is taken into account. A significant difference between the results presented in Ref. 21 and in the submitted paper lies in the estimated effect of the domain wall on the barrier height for Schottky emission which is much stronger according to Ref. 21 than according to the submitted paper. At least a brief discussion of the similarities in and differences between the results (and possible reasons for them) should be presented.

We thank the referee for this comment. The assignment of the specific conduction mechanism is a notoriously difficult task given the uncertainties and variations between the samples. However, we want to reiterate that the main point of our manuscript is that the constitutive properties of the transport junction will be defined by the *field-induced* polarization configurations, rather than as grown ones. Therefore for each specific case and sample there should in general be a substantial range of junction parameters, and the breadth and evolution of this range with applied field define the conduction properties. In this case, the IV curve can be fit for a single set of parameters only in a narrow range of applied voltage.

As for the specific comparison to the Noheda group's work, we note that the space-charge limited conduction (SCLC) behavior typically results in slopes in the log(J) vs log (E) representation of 2 (Mott-Gurney) as observed in the paper in question[2], but can be higher in the case of a distribution of traps.[3] We have conducted further fitting, which is illustrated in supplementary S3, and shows that the slopes we calculate for the SCLC behavior are between 3

and 4. However the residuals are slightly higher than for Schottky emission. Given this, we have gone with the Schottky emission fits, although this is now discussed in the main text on page 7-8. It further suggests that the distribution of defects is likely different in this film as compared to that of the Noheda group, but as we note above, this is rather unsurprising.

The authors write, "Strikingly, the domain walls in this ultra-thin BFO sample display conduction, a result which has not been reported in ultra-thin ferroelectric films to date." The question is whether such results have not been reported because no studies were performed on ultra-thin BFO films, or whether the studies were conducted but did not demonstrate any conductivity enhancement by domain walls. If the former is the case, then the conductivity effect found in the submitted paper should not be surprising, given numerous data on the domain wall effect on conductivity in thicker films and even in bulk material. If the authors of the submitted paper assume that 10 nm films should be significantly different from 30 nm films in that respect, then the reasons for such an assumption should be provided.

We have reworded it such that we state that this is the first report of the phenomena in ultra-thin films (page 6, “The domain walls in this ultra-thin BFO sample display conduction, and as such is the first report of this phenomena in ultra-thin ferroelectric films (though not entirely surprising”). Indeed, we expect to attempt to reduce spreading of current into bulk using thinner films, to better separate the wall vs. domain.

The authors stress several times that the discovered twisted domain wall configuration has a non-zero polarization curl which "is a situation that is classically forbidden by Maxwell’s laws for electrostatics". Maxwell's laws prohibit only a non-zero curl of polarization induced by the applied electric field (as an electrostatic field itself has a zero curl according to Maxwell’s laws). Permanent dipoles produced in a ferroelectric may form a polarization field with a non-zero curl by forming vortex shaped polarization patterns as has already been demonstrated both experimentally and theoretically.

We take the point that the non-zero curl is forbidden for the applied electric field, but is possible for the polarization field, and have therefore clarified these statements in the manuscript.

Some of the figures need improvement. In Fig. 2.b, the barrier height and the high-frequency dielectric constant values are presented, however, it is almost impossible to read them. Fig. 6 presents polarization patterns in twisted domain structures, however, they are so small that it is not possible to understand the polarization distribution in them. These problems may be solved if the twisted structures are cropped from a larger domain pattern and presented with a larger magnification. Alternatively, the figures could be moved to the "Supplementary information" section where they could be presented in a larger size.

We have greatly modified figures 2 onwards in the revised manuscript. The twist patterns are now much larger in size to better observe the polarization distributions. We have also substantially improved our fitting procedure to account for the noise-floor of our current amplifier, and obtained an even better agreement with the Schottky barrier model, as described below.

Reviewer #3 (Remarks to the Author):

A new twist on domain wall conduction by Rama K. Vasudevan et al. reports on the origin of conduction at uncharged domain walls, namely 71o domain walls, in BiFeO3 films. The authors use more sophisticated techniques than previous reports to confirm: a) the mechanisms for conduction of domain and domain walls are the same, that is there is not intrinsic domain wall conduction origin, such as a lower band gap and b) the mechanism is Schottky emission and, thus interface determined. Next to the more robust and statistically more significant data analysis using the PCA noise reduction method, the added value of this paper is to show that conduction of domain walls is also present in films as thin as 10nm.

We appreciate the referee's acknowledgement of our robust statistical analysis and for the succinct summary of the findings, and thank them for their comments.

But the original idea of the paper that justifies its consideration in a high impact journal such as Nature Comm. is the proposal that the increased currents at domain walls are due to locally enhanced electric field, as opposed to locally decreased values of the Schottky barrier due to migration of space charge to the walls, as previously proposed. This argument is supported by phase field simulations showing that polarization curls could form at the domain walls and could eventually amplify the electric fields sufficiently.

We thank the referee for their comments, and appreciate that they note the evidence provided for the polarization curl mechanism from phase-field simulations

Even though I think it is a very interesting idea, certainly worth to be published, I doubt that this journal is the proper place for it, since evidence of those polarization curls is not shown.

Coming to the criticism, direct evidence of polarization curl under an applied SPM tip is near impossible with existing instrumentation in a conducting AFM experiment. However, curl-like motifs were already invoked in earlier studies of lowered nucleation bias at wall[4], hysteretic conduction in $BiFeO_3$[1] as well as direct imaging of larger twist structures[5]. Our main emphasis here is that at the energy scales involved in conductive AFM experiments, polarization reconfiguration is already very facile. The curl motif arises as a result, and it provides a consistent accounting of the observed domain wall conduction, as a field-induced modification of the domain conduction.

The authors write that this proposed mechanism does not need the presence of defects, but it is a fact that those defects are present in the films and it has been demonstrated by several authors cited in the manuscript that changes in the defect concentration (either by oxygen vacancies or

by cations) give rise to clear changes in conductivity. So defects need to be taken into account when simulating the electrostatic potential with or without curls.
To this point, it is of course conceivable that the energies will be sufficient to create defects also. The primary driving force will again likely be the curl motif, where defects would screen the polarization and reduce the divergence, thereby reducing the degree to which the field enhancement occurs. However, this takes time, and the polarization will also evolve to accommodate such changes (they are fully coupled sub-systems). The exact interplay between defects and the polarization curl is a complex topic and warrants more study by phase-field simulations, but we believe is beyond the scope of this paper. We therefore would leave such further simulations and analysis to a subsequent manuscript. We note that basic simulations of oxygen vacancies show that the electrostatic potential step at the pre-existing domain wall is somewhat reduced by screening from oxygen vacancies, as shown below in Figure R1, but the effect is small.

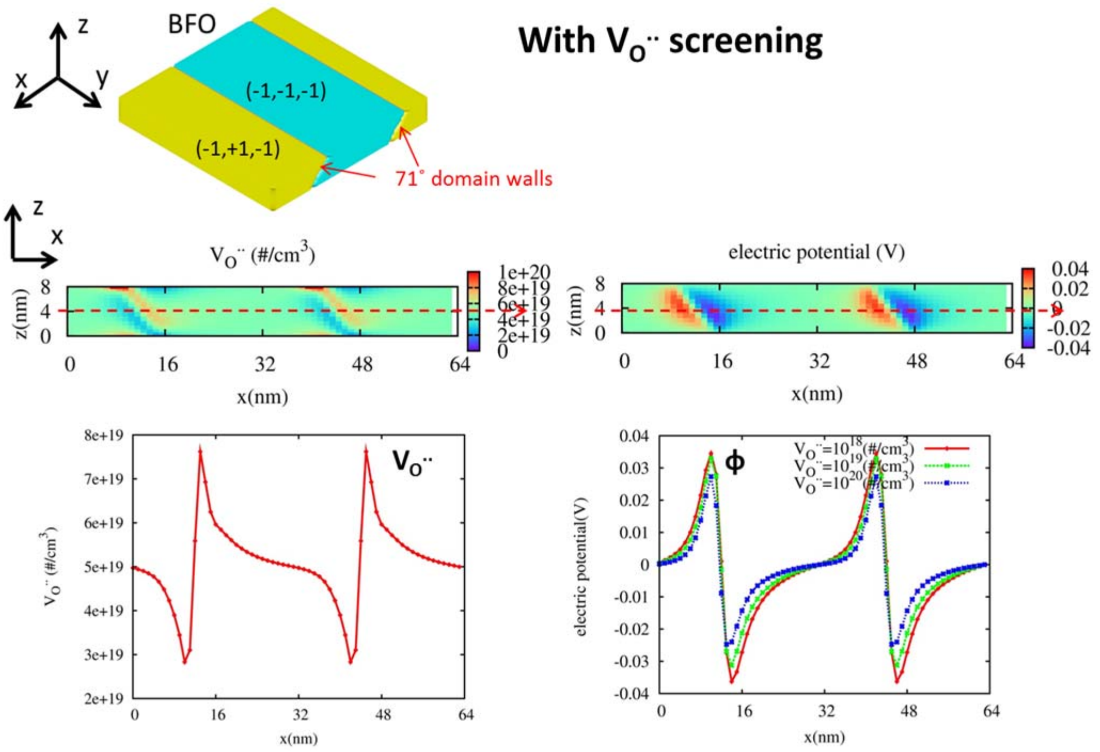

Figure R1: Phase-Field simulations including oxygen vacancies, showing their effect on the potential step around a 71° domain wall. The increasing concentrations serve to slightly reduce the potential, and the vacancies segregate somewhat to the surface, but the main features of the potential profile are not dramatically altered.

The effect shown in Figure 5 is not convincing enough (differences in slope with the experimental data are assigned to field non-linearity, which will certainly play a role but it is not clear in which direction and how). At the very least the simulations should show the comparative effect of the decrease in Schottky barrier upon defect migration and maybe also polarization re-orientation (effect that one cannot ignore) and the increase of the electric field.

We have performed additional phase-field simulations to directly measure the degree of field enhancement as a function of applied potential, as shown in the new Fig. 6(a), where the nonlinearity of the field enhancement is strikingly evident. In the simulations, it appears to increase until about 1.8V, but decreases thereafter. This means that we expect to observe higher slope after the field enhancement effect becomes appreciable. However, this becomes complex if one considers transition of specific conduction mechanisms at some particular bias, as has been observed by previous authors. Moreover, the field enhancement should reduce with increasing voltage applied to the tip and the slope will return to near the original value. As an example, we plot below a simulated I-V curve following Schottky emission regime (blue) as well as the same curve, in the nonlinear field enhancement case (red).

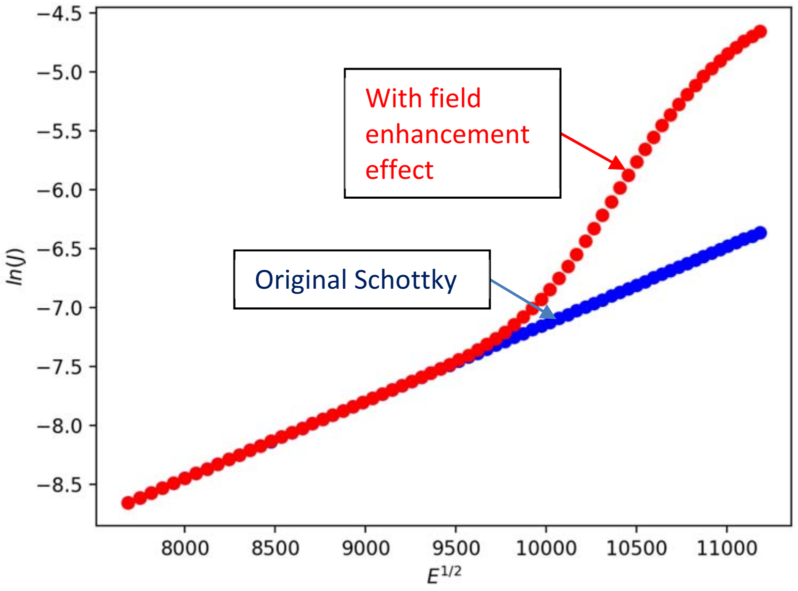

Other points of consideration are:

1) the fit to the Schottky emission I-V curves gives high-frequency dielectric constants of 40-50 which are much larger than the expected values (around 5). In fact the simulations use very similar value for the static dielectric constant.

In our revised manuscript we revisited the I-V data, and found that there was a small offset in the current at 0V which arises from the instrument, that should have been subtracted. Moreover, as referee 4 points out, there exists a kink in the I-V behavior that we investigated. The result of these new analyses are now shown in Figure 2, and are also shown below. The first important fact to note is that if we simply plot the IV curve from the domain wall vs. the IV curve taken on the domain, and then plot the wall current divided by the domain current, we notice that the response is constant, strongly suggesting the same mechanism is at play (Fig. R2(b)).

Moreover, we find two linear regimes in many points in the spectroscopic map (Fig. R2(a,b)). We fit each I-V curve to a linear equation in the Schottky emission representation (ie., Log J vs $E^{0.5}$), and further fit the same curve to a two linear segment fit. We then compute the Bayesian

information criterion[6] which allows determination of which model choice is more appropriate for the given data. If the single linear fit is more appropriate at that point, we use those values in the determination of the Schottky parameters (barrier height, dielectric constant) corresponding to that point. Else, we have sets of parameters corresponding to both the first and second fitting segments that differ. The results of the fit are shown below in Figure R2. (e-h) for the first and second segments, respectively. The threshold voltage map plotted in Fig. R2(d) shows the voltage of the transition between the first linear segment and the second linear segment, in pixels (points) where the BIC determined the two linear segment fit was the more appropriate model. Many points at the domain wall transition to another mechanism at high voltage; however, as the transition occurs at relatively high voltage, the small number of datapoints precludes further analysis of the conduction mechanism for this segment. Still, the existence of the kink in the I-V behavior is suggestive of the field-enhancement mechanism.

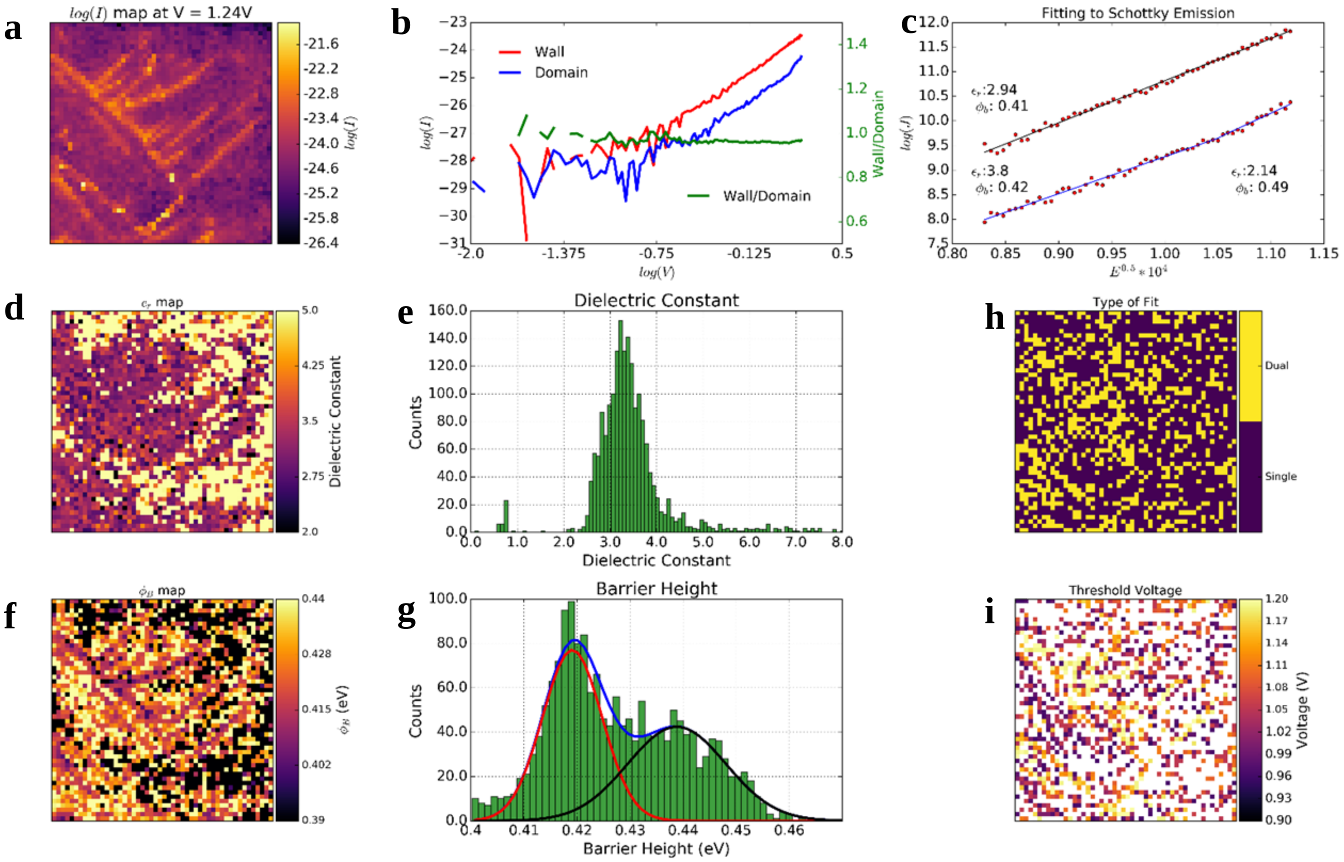

Figure R2: Redone I-V analysis, with double linear fitting. (a) log(I) map at V=1.24V from the I-V spectroscopic experiment (the voltage is applied to the sample). Two representative points from the domain and domain wall are shown in **(b)** in a log-log scale. The wall current/domain current is plotted on the right-side y-axis, and shows near constant values indicating same mechanism. **(c)** Two example fits from the data, plotted in the Schottky representation. For one of the points, the fit is best described by a single line fit (black line). The extracted parameters for the dielectric constant and the barrier height are indicated alongside the data. For other points, a two-segment fit is more appropriate (blue line). Barrier height and dielectric constant for both segments are indicated (left: first segment, right: second segment).

(d,e) Spatial map and histograms of the dielectric constant , and **(f,g)** spatial map and histograms for the barrier height, computed as discussed in the text. It should be noted that for points where the singe linear equation is preferred to the two linear segment fit, the values in these maps correspond to the fitting from the single linear equation; else they correspond to the first segment fit. **(h)** Bayesian information criterion allows assessment of which model (single or dual linear segments) is more feasible; this is mapped spatially. **(i)** For those points which can be best fit by two linear segments, the threshold voltage (from the first segment to the second segment) is plotted.

2) the simulations use a tip of 5 nm (radius?) while the AFM metallic tips are typically 3 times bigger.

In the phase-field simulation we modeled the AFM tip by defining the electric potential distribution on film surface using a Lorentz function (see Eq. (S-3) in the supplementary), and γ=5nm is the half width half magnitude (HWHM), i.e., the width from the tip center where the electric potential decreases half of its maximum at the tip center (see Eq. (S-3)). So γ is not the radius of the AFM tip, which is typically ~20nm. We also used γ=8nm and γ=10nm in modeling AFM tip in the phase-field simulation and it does not greatly affect the results of the simulation. Therefore, we believe it is reasonable to use γ=5nm in the current simulation.

3) The samples are 10nm thin while the simulations consider a 32 nm thick film. Why not the same thickness?

We recomputed the simulation for the new manuscript, taking care to use a 10nm film in this case.

4) In general the relevant papers are cited but not always in the proper way. For example: “The case of enhanced conductivity at the nominally uncharged ferroelectric domain walls was a particularly surprising finding, as good ferroelectrics are semiconductors with wide bandgaps (>~2.5eV). “

Reference [11] should be cited after “finding“ as it was the first report on domain wall conductivity.

We take the referee’s point and have referenced the papers in a more correct fashion, consistent with the initial reports of conducting behavior as well as the mechanism of Schottky emission.

Another example is also mentioned above: this paper confirms a) the mechanisms for conduction of domain and domain walls are the same and b) the mechanism is Schottky emission and, thus interface determined. Both were reported by the Groningen group against the, then, prevalent models. The way these papers are cited does not properly reflect this fact. E.g. In “To identify the conduction mechanism, we began by attempting to fit the I-V curves to various conduction models. Of all the mechanisms investigated (Schottky emission, Poole-Frenkel, space-charge limited conduction, Fowler-Nordheim (FN) tunneling and variable range hopping), only Schottky emission returned reasonable results.“ one misses “as reported in ref.21”.

We have added this remark as suggested in the revised manuscript.

Reviewer #4 (Remarks to the Author):

Vasudevan et al. address domain wall conduction in terms of conductive atomic force microscopy and phase-field simulations. The work contains several new and interesting ideas / results, but additional data is required in order to support the conclusions drawn in the manuscript. After the authors provided such data and improved the discussion, the manuscript might be of interest for the readership of Nature Communications.

General:

1. The manuscript contains some typos, e.g., line 96 should read “domain walls” not “domains”, line 127: “one is missing”, etc.

We have proof-read our revised manuscript to remove these grammatical and typographic mistakes.

2. Some references need to be added, e.g.; line 51/52: band gap for “good ferroelectrics”. Line 94/95: “this is precisely the situation incurred during c-AFM...”.

We have added references for these statements.

Scientific:

1. The authors write that “only Schottky emission returned reasonable results” (line 143). Fitting of the other relevant models, including a discussion of the fitting constants, should be presented in the supplementary. This way the reader can understand why the model is the only reasonable one, which is quite often not clear in cAFM. I suspect, for instance, that the thermionic emission model by Simmons can describe the data, too. Thus, the presentation of the analysis should be improved.

We thank the referee for this suggestion, have included a new section in supplementary S3, where we illustrate fits from the various possible conduction mechanisms. The results indicate that the two most plausible mechanisms are Schottky-emission and space-charge limited current (SCLC), but the SCLC slopes are somewhat high (as expected for samples with a distribution of trap states[3]). Given the fact that the residuals are slightly higher than for SCLC than for Schottky emission, we have utilized Schottky. But as we explain to referee 2, the exact determination of the conduction mechanism is notoriously difficult, and instead we aim to focus our attention on the proposed mechanism of the field enhancement, which will be applicable regardless of the specific bulk or interface-limited current mechanisms at hand.

2. Data for positive and negative bias should be shown in Fig. 2. This could further support the interpretation of the conduction mechanism presented in the paper.

Our data shows that at negative sample bias, there is no conduction until very high voltage is applied, after which there is breakdown. An example bipolar I-V curve is shown below. This

rectification is not surprising given the barrier. Three such I-V curves obtained on the same BFO sample in UHV conditions are shown below in Figure R3.

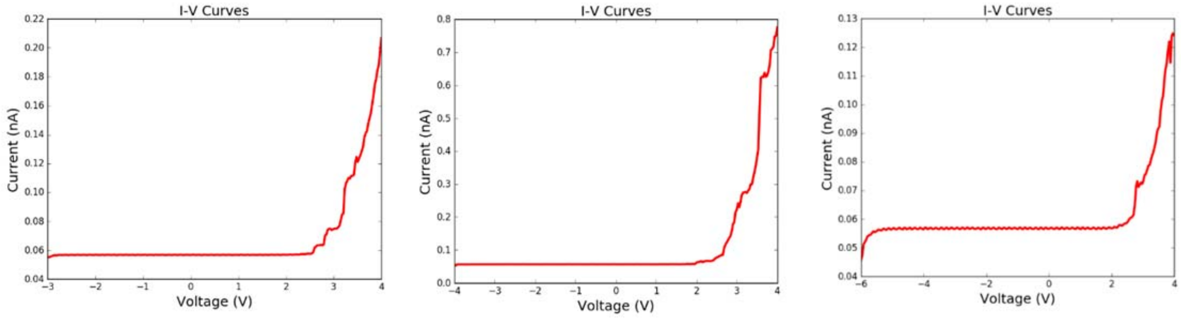

Figure R3: Bipolar I-V curves on the BFO sample, showing that almost no current can be measured for V_{sam}>-4V on the negative side.

3. Moreover, fits for the domain and the domain wall should be shown in Fig. 2c. It is said that domain and wall exhibit the same conduction mechanism. The domain data, however, shows an intriguing kink that should be addressed.

We thank the referee for pointing out this feature in the I-V curves, which we have now studied in detail. Please see earlier response above.

4. The voltage applied during the cAFM scan is about 5 order of magnitude larger than in previous studies performed on, e.g., LiNbO3 or HoMnO3 bulk materials. Thus, defects at the domain wall and electrical breakdown may start playing a crucial role for the observed conductance. This should be discussed, especially as no intrinsic effect such as the reported twist would be needed to explain the experimentally observed phenomena.

We take the point that the field applied by the tip can be rather large compared with macroscopic contacts, and approach those of breakdown. However, we do not see evidence of breakdown at such applied potentials, such as irreversible topographic changes. This is further evidenced by overall reproducibility of IV curves. Therefore, for the time-being, we consider only the changes in the polarization profile in applied electric fields. We do agree that at these fields, mobile defects may also begin to redistribute. However, we will only be sensitive to such changes above a certain threshold for defect density, which may either occur at higher voltages or in more defective samples (as is generally the case with memristor materials).

5. Since dynamical phenomena are discussed, time-dependent cAFM data could provide additional insight and support the authors interpretation.

This is true. We performed some basic measurements where the slow-scan axis was disabled for some time, and the area was re-scanned. This experiment is in the supplementary, and is shown here for clarity in Fig. R4.

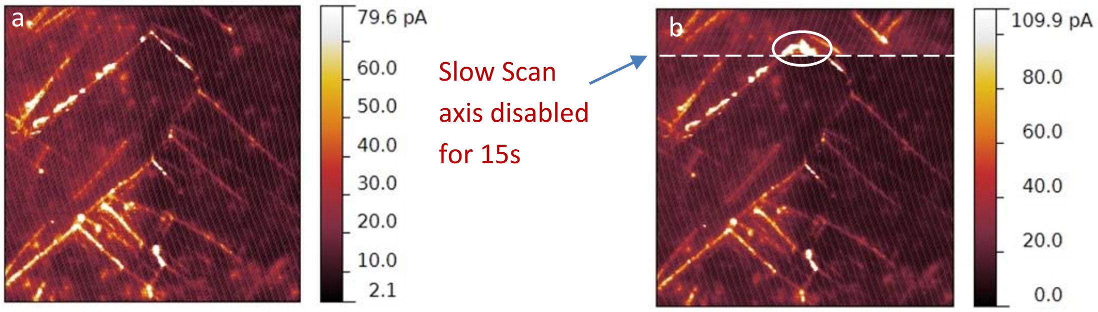

Figure R4: Conductivity change by repeated scanning. (a) c-AFM with Vsam = +1.2V. During the subsequent scan, the slow scan axis was disabled for 15s at the dashed line. The c-AFM scan after this procedure is plotted in (b) and clearly shows that the junction in the center of the line (circled) appears to have increased conductivity compared to (a).

We note that with time, the walls seem to show a somewhat larger conductivity. This is likely due to migration of defects, and possible stabilization of the twisted structure

We note that migration of defects. vs stabilization of twist structure with time can both occur, but that both polarization and mobile defect sub-systems would be strongly coupled.

6. Moreover, direction dependent scans (forward and backward) should be presented in Fig. 1. Based on the simulations, I would expect them to look substantially different on 71-degree domain walls.

We have observed a slight difference in conduction based on scan direction; this is highlighted in this image below in Fig. R5, which has been added to the supplementary S6. In addition to higher current on the forward scan direction (as compared to the reverse scan direction), the conduction appears to be non-Gaussian at the walls, providing further evidence for this mechanism.

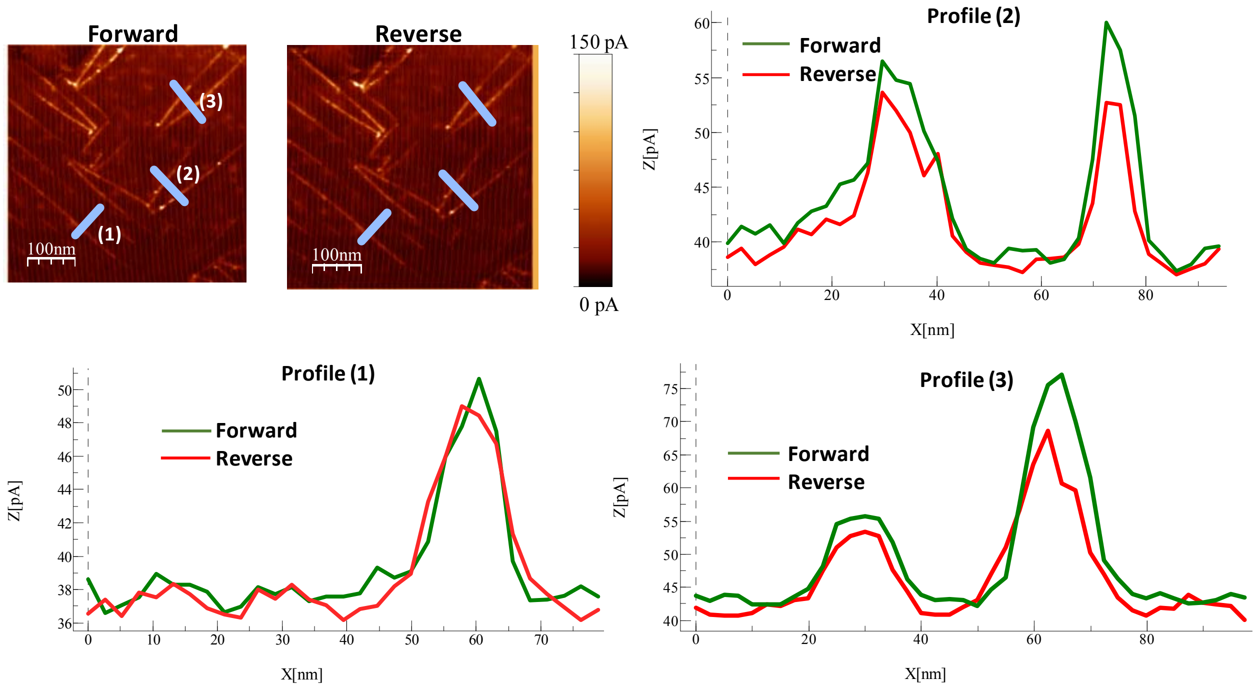

Figure R5: c-AFM scans with V_{sam} = +1.2V. The forward and reverse scans have been aligned to facilitate appropriate comparison of line profiles. The line profiles are averaged over 5 pixels for noise reduction. The forward scans tend to indicate higher conduction than reverse direction scans, and furthermore, the conductivity around the domain walls is asymmetric – both of which would be predicted by the wall twist mechanism.

7. What is the explanation for the lower dielectric constant observed at the domain walls in Fig. 3a?

As explained above, our new fitting with the two linear segments, and accounting for the instrument offset at 0V, seems to solve these issues, and now little contrast can be seen between the domains and the domain walls.

8. The simulations in Fig 4 are performed for significantly thicker films than measured by cAFM. Simulation results in the thin film limit should be presented and discussed in order to make the connection to the experiment.

We repeated the simulations for a 10nm case, instead of 32 nm, to better match the experimental conditions. These results are in the revised manuscript.

9. I do not fully understand why the twisting caused within a domain (Fig 4c, first panel) does not involve enhanced currents in cAFM? It would be helpful to elaborate this in more detail.

This is a very crucial point which we have tried to communicate better in the revised manuscript. The formation of the polarization nucleus occurs both within the domain and at the domain wall. However, the existence of the domain wall simply allows the twisted structure to form at lower

fields. Its detailed structure is also subtly different. The lower threshold has the primary effect that at any given voltage, the "twisted" nucleus is larger for the domain wall than the domain, and also has greater field enhancement at the interface according to phase-field modeling. Therefore, the wall appears to be more conducting. In other words, the presence of topological defects in polarization field facilitates bias-induced polarization dynamics.

That said, the referee is correct in that even within the domain, there is a field enhancement from this nucleus structure, if one compares it to the case of a linear dielectric where no such nucleus structure will form. Indeed, we compared the polarization and field enhancement behaviors in a linear dielectric and a single domain ferroelectric in a prior publication.[7] We have tried to better explain this point in the new manuscript. We have also explored the field enhancement in more detail (as a function of applied potential), with results plotted in the new Figures 4-6. The main finding is reproduced below in Figure R6, where we note the field enhancement peaks at a particular applied potential.

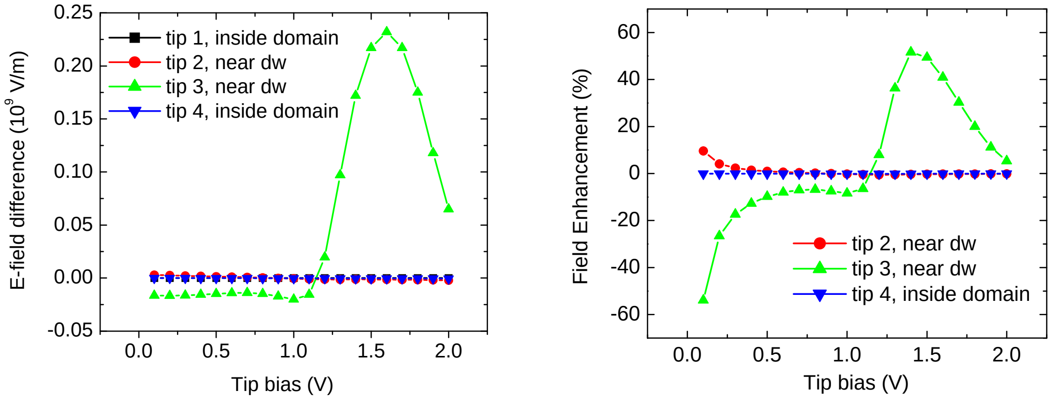

Figure R6: Phase-field derived field differences and field enhancement for tip at the domain wall vs. within the domains, as a function of tip bias. The field enhancement is decidedly nonlinear, peaking at about V = 1.7V.

References

1. Maksymovych P, *et al.* Dynamic Conductivity of Ferroelectric Domain Walls in BiFeO3. *Nano Lett* **11**, 1906-1912 (2011).

2. Farokhipoor S, Noheda B. Conduction through 71 degrees DomainWalls in BiFeO3 Thin Films. *Phys Rev Lett* **107**, (2011).

3. Rose A. Space-charge-limited currents in solids. *Physical Review* **97**, 1538 (1955).

4. Balke N, *et al.* Deterministic control of ferroelastic switching in multiferroic materials. *Nat Nanotechnol* **4**, 868-875 (2009).

5. Vasudevan RK, *et al.* Exploring Topological Defects in Epitaxial BiFeO3 Thin Films. *Acs Nano* **5**, 879-887 (2011).

6. Wit E, Heuvel Evd, Romeijn J-W. 'All models are wrong...': an introduction to model uncertainty. *Statistica Neerlandica* **66**, 217-236 (2012).

7. Cao Y, Ievlev A, Morozovska A, Chen L-Q, Kalinin S, Maksymovych P. Intrinsic space charge layers and field enhancement in ferroelectric nanojunctions. *Appl Phys Lett* **107**, 022903 (2015).

<u>Reviewers' Comments:</u>

Reviewer #1 (Remarks to the Author):

In the reply to the comments 2, the authors state that "The key differences are that we posit that the domain wall becomes conducting through essentially field-induced excitation of the polarization order parameter, and therefore its conductivity is a transient and dynamic effect." It suggests the new finding of the present work is that the domain wall conductivity is a transient and dynamic effect compared with the previous work [Nature Communications|4:1808|(2013)].

However, in the reply to comments 3, the authors state that "It is true that here we consider the perturbed domain states to be quasi-static for the given potential. Hence, in this case we deal with the voltage-induced polarization responses, rather than time dynamics." The quasi-static process is not consistent with the transient and dynamic effect as stated above. If the polarization evolution is quasi-static, what's the difference of the present domain conductivity from previous ones? From the physical point of view, the mechanism of conductivity of twist domain wall is the same as that of previous head-to-head or tail-to-tail domain walls.

Reviewer #2 (Remarks to the Author):

As I wrote in my review of the original submission, the submitted paper presents new and very important results and deserves to be published. The revisions implemented by the authors have significantly improved the quality of the paper and responded to most of the reviewers' criticism. I think that the submitted paper adds important data to the study of the domain wall conductivity. In my opinion, the main strength of the paper is the combined use of an experimental study and phase field modeling. The results of this study indicate that there may exist a new mechanism for the effect of the domain wall on the conductivity of thin ferroelectric films, i.e., an enhancement of the conductivity through an applied field induced polarization nucleus causing the warping of the domain wall.

I would like to note that I agree with one of the reviewer's comment that this effect may be of a quasi-static nature and that the role of the dynamics of the domain wall movement can be confirmed only if dynamic simulation results or experimental results, which demonstrate the effects of the domain wall/polarization nucleus dynamics, are presented. The authors confirm in their response, "... here we consider the perturbed domain states to be quasi-static for the given potential. Hence, in this case we deal with the voltage-induced polarization responses, rather than time dynamics." Therefore, it may be more suitable to use, "the field induced domain wall perturbations" or "the field induced domain topological defects" instead of "the dynamic

topological defect" or other similar dynamic effects.

Reviewer #3 (Remarks to the Author):

The manuscript has improved drastically with the re-fitting of the I-V curves and the additional clarifications. I can now advise its publication in Nature Communications.

Response to reviewers

Reviewer #1 (Remarks to the Author):

In the reply to the comments 2, the authors state that "The key differences are that we posit that the domain wall becomes conducting through essentially field-induced excitation of the polarization order parameter, and therefore its conductivity is a transient and dynamic effect." It suggests the new finding of the present work is that the domain wall conductivity is a transient and dynamic effect compared with the previous work [Nature Communications|4:1808|(2013)].

However, in the reply to comments 3, the authors state that "It is true that here we consider the perturbed domain states to be quasi-static for the given potential. Hence, in this case we deal with the voltage-induced polarization responses, rather than time dynamics." The quasi-static process is not consistent with the transient and dynamic effect as stated above. If the polarization evolution is quasi-static, what's the difference of the present domain conductivity from previous ones? From the physical point of view, the mechanism of conductivity of twist domain wall is the same as that of previous head-to-head or tail-to-tail domain walls.

We appreciate the reviewer's attention to this important aspect. The core of our proposal is that the apparent domain wall conductance is due to metastable, field-induced topological defect – which is a composite of intersecting domain walls in a small nanoscale volume. Although the meta-stability of these "field-induced topological defects" is assumed to persist over some finite voltage window of the I-V curve around the measurement points, it doesn't have to be, in general. The relaxation of the specific field-induced configuration may be faster or slower than the acquisition rate of the I-V curves. We believe that this metric would differentiate between "dynamic" and "quasi-static" topological defects. To avoid confusion for the time-being, we drop the word "dynamic" in this manuscript and just refer to it as "field-induced topological defect".

We further emphasize that the mechanism by which these metastable entities enable finite conductance is charge screening in the contact region, qualitatively similar to space charge in semiconductor contacts. Strong carrier accumulation at the domain walls themselves, and their transition to metallic state assumed in prior works is not required. Of course, such "surface gating" and "bulk gating" effects may coexist depending on specific measurement and material cases.

We have rephrased the terminology in the paper to refer to "field-induced topological defect", and drop the word "dynamic".

Reviewer #2 (Remarks to the Author):

As I wrote in my review of the original submission, the submitted paper presents new and very important results and deserves to be published. The revisions implemented by the authors have significantly improved the quality of the paper and responded to most of the reviewers' criticism. I think that the submitted paper adds important data to the study of the domain wall conductivity. In my opinion, the main strength of the paper is the combined use of an experimental study and phase field modeling. The results of this study indicate that there may exist a new mechanism for the effect of the domain wall on the conductivity of thin ferroelectric films, i.e., an enhancement of the conductivity through an applied field induced polarization nucleus causing the warping of the domain wall.

We thank the reviewer for his positive remark.

I would like to note that I agree with one of the reviewer's comment that this effect may be of a quasi-static nature and that the role of the dynamics of the domain wall movement can be confirmed only if dynamic simulation results or experimental results, which demonstrate the effects of the domain wall/polarization nucleus dynamics, are presented. The authors confirm in their response, "... here we consider the perturbed domain states to be quasi-static for the given potential. Hence, in this case we deal with the voltage-induced polarization responses, rather than time dynamics." Therefore, it may be more suitable to use, "the field induced domain wall perturbations" or "the field induced domain topological defects" instead of "the dynamic topological defect" or other similar dynamic effects.

We agree with the reviewer, and we have reiterated this point in response to reviewer 1. Indeed, we have changed the terminology to the “field-induced topological defect”.

Reviewer #3 (Remarks to the Author):

The manuscript has improved drastically with the re-fitting of the I-V curves and the additional clarifications. I can now advise its publication in Nature Communications.

We thank the reviewer for his remarks and recommendation.
