# Supplementary material for: Field enhancement of electronic conductance at ferroelectric domain walls
Source: Nat Commun. 2017 Nov 6;8:1318. doi: 10.1038/s41467-017-01334-5 (PMC5673066; doi:10.1038/s41467-017-01334-5)
Supplement: Supplementary file 2 — Peer Review File [file 41467_2017_1334_MOESM2_ESM.pdf]

Supplementary Note 1 – X-ray Surface Diffraction and Microscopy

Surface Diffraction Hard x-ray diffraction studies were performed at Sector 33 ID-D of the Advanced Photon Source. Crystal truncation rods of $DyScO_3$ ($00L_{pc}$, $10L_{pc}$, $\bar{1}0L_{pc}$, $11L_{pc}$, $1\bar{1}L_{pc}$, $\bar{1}10L_{pc}$), where L is the Miller index, were measured to probe the epitaxial relations between the $BiFeO_3$ and the substrate. The well-pronounced thin-film Kiessig fringes in Supplementary Figure 1a indicate the good structural coherence of between the Bragg planes of the film and the substrate and were used to estimate the film thickness to be ~ 30 unit cells. In addition, a rocking curve as a function of the Bragg angle was measured at the $BiFeO_3$ 002_{pc} reflection (Supplementary Figure 1b). By fitting the rocking curve to a Gaussian curve, we extracted a FWHM of ~ 120 arcsec, indicating high crystal quality (substrate's FWHM is 12 arcsec). From the Bragg peak positions of $BiFeO_3$, the lattice parameters of the film were refined in a monoclinic unit-cell, obtaining $a = 5.616 \pm 0.005$ Å , $b = 5.615 \pm 0.005$ Å, $c = 3.990 \pm 0.005$ Å, $\alpha = \gamma = 90°, \beta = 89.63°$. The in-plane (orthorhombic) lattice parameters of the substrate are $a = 5.454$ Å and $b = 5.723$ Å.

Diffraction Microscopy Hard x-ray diffraction microscopy (XDM) was used to image the ferroelastic domain configurations of $BiFeO_3$ with sub-100 nm lateral spatial resolution.[1] The imaging contrast in XDM emanates solely from diffraction and can be used to distinguish between 71° domains (bright contrast) and other domain configurations (e.g. $109\ ^o$) that appear with dark contrast in the XDM image (Supplementary Figure 1c). This data shows that in this $BiFeO_3$ thin-film 71° domains are the dominant configuration, in agreement with PFM measurements, and justify the initial domain configuration used in the phase-field simulations.

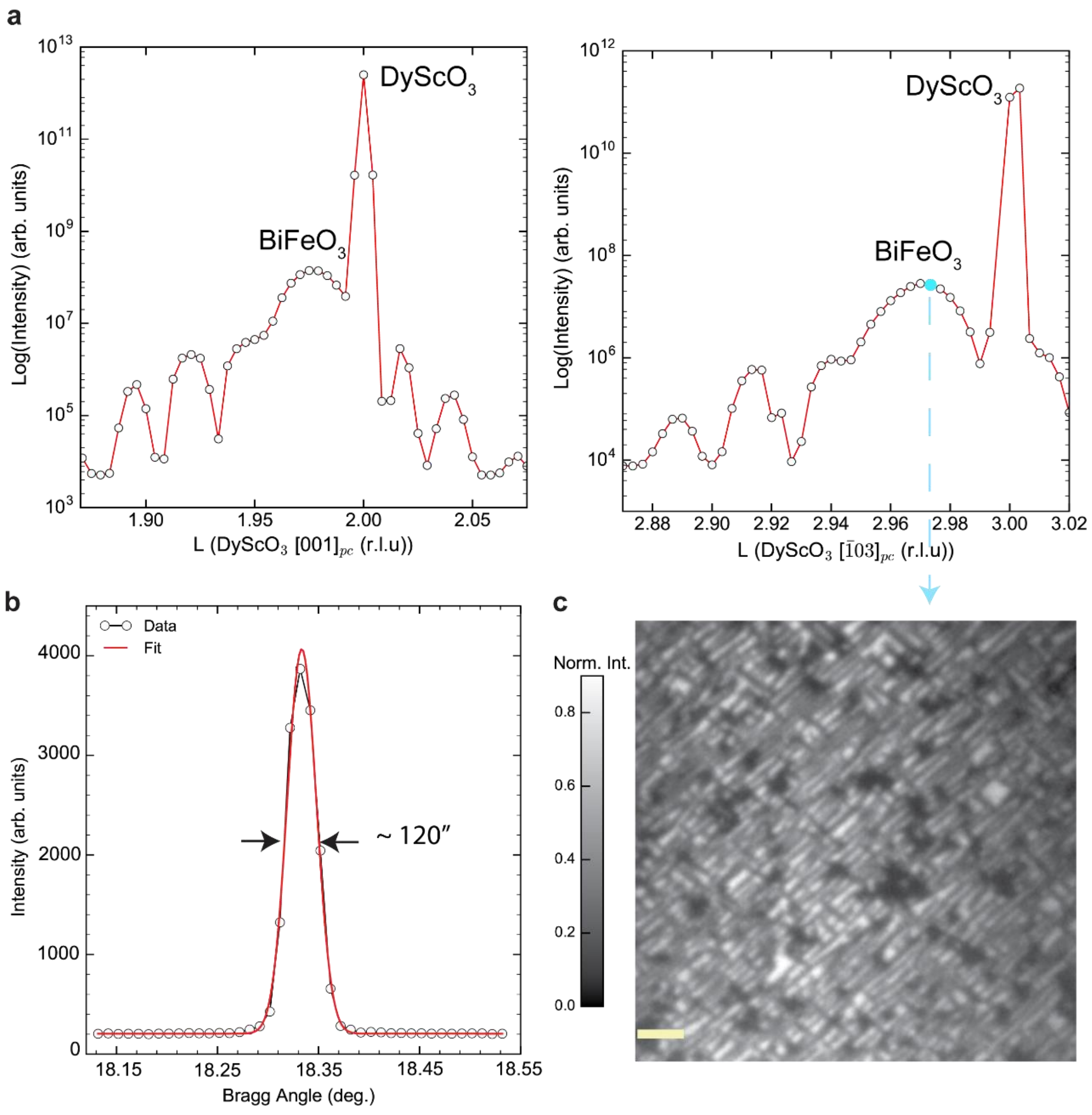

Supplementary Figure 1. X-ray Diffraction Scattering and Microscopy of $BiFeO_3/DyScO_3$. (a) Close-ups of the $00L_{pc}$ and $\bar{1}0L_{pc}$ rods of $DyScO_3$ near L=2 (right panel) and L=3 (left panel) indicate that the film is coherently strained to the substrate in both in-plane and out-of-plane crystallographic directions. **(b)** Rocking curve taken at $BiFeO_3$ 002 reflection is fitted to a Gaussian curve to extract a FWHM of 120 arcsec, which indicate a film with good crystal quality. **(c)** X-ray diffraction microscopy image was acquired at a scattering vector $\mathbf{q}_{HKL} = \mathbf{q}_{\bar{1},0,2.97}$, as indicated by the arrow in (a). Bright regions indicate domains with 71° walls and dark regions indicate ferroelastic domains with 109° walls. The scale bar in (c) is 1 μm and the intensity is normalized diffraction contrast.

Supplementary Note 2 – PCA Denoising of the I-V Data

To fit the various conduction models to the data, the data was first denoised to eliminate the large 60Hz oscillations present (due to the current amplifier). We utilized principal component analysis, performed via the singular value decomposition (SVD) method to conduct this denoising. The SVD method factorizes a matrix *M* (of size *m* x *n*, in this case, position index by voltage step):

$$M = USV^*$$

where *U* is an *m* x *m* unitary matrix, *S* is a diagonal matrix of size *m* x *n* and V^* is an *n* x *n* unitary matrix. The eigenvectors (components) are given by the columns of V and the columns of US are the scores ('loadings'). PCA[2] is a method that decomposes based on variance in the data, such that the first component contains the most variance, the second component contains the second most variance, and so on. In practice, higher order components generally contain only noise, allowing PCA to be used effectively for denoising purposes. Plots of the results of SVD for *the I-V* spectroscopic dataset (size 2500 x 128) are shown below in Supplementary Figure 2. Remarkably, the noise is isolated as separate components during the decomposition. The data could then be reconstructed based on removal of these components (to reform the matrix *M*, utilizing only the first *k* principal components, thereby avoiding higher order terms that consist mainly of noise). In this case, we set *k* = 7, after removing components 3 and 4 from the data. We further denoise by smoothing of components 5-7, to remove the associated 60Hz oscillations, using a basic averaging filter. The results of the denoising of these individual components is seen in Supplementary Figure 3. After denoising of these components and removal of components 3 and 4, the data (up to k=7) was reconstructed to complete the process. We also removed the current offset at 0V before analysis of the I-V curves.

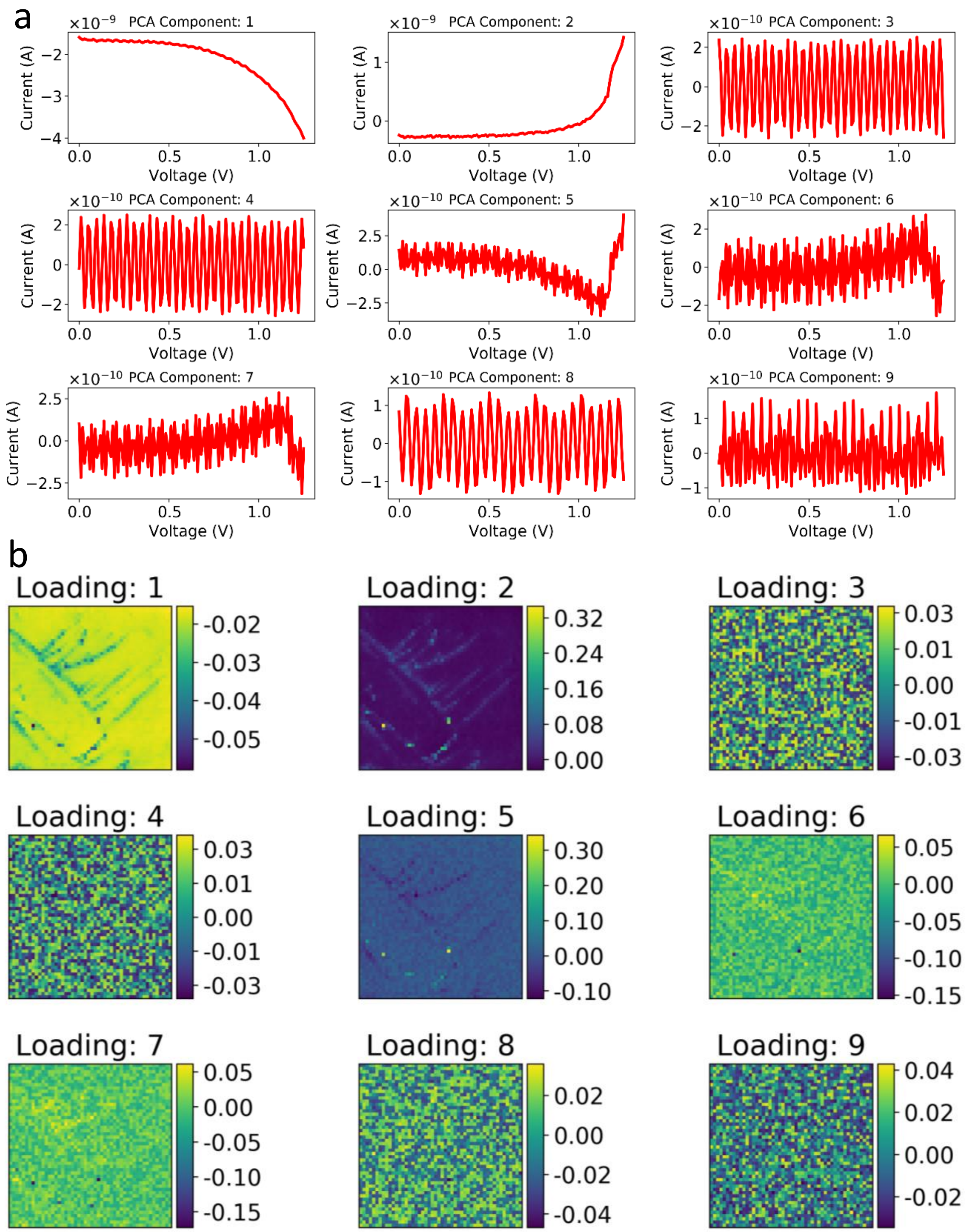

Supplementary Figure 2: PCA-based denoising of data. (a) First 9 PCA Eigenvectors and **(b)** Loading maps from SVD-based analysis of the I-V spectroscopic data.

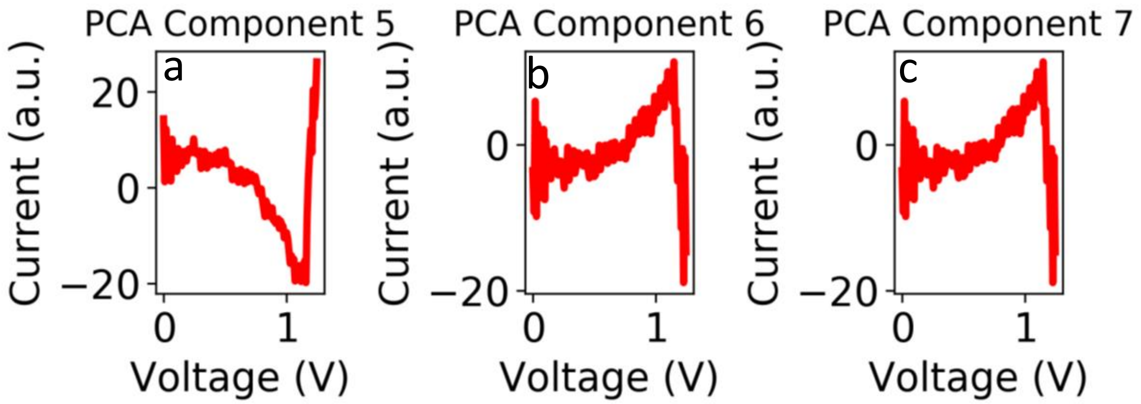

Supplementary Figure 3: Averaging filter on PCA components 5-7. Each individual component **(a-c)** was subjected to a basic averaging filter to smooth the oscillations present. The results of applying the filter are shown above (the filter was only applied to these components; other components were not adjusted).

Supplementary Note 3 – Schottky emission fits and other mechanisms

Various conduction mechanisms were tried, including Variable Range Hopping (VRH), Poole-Frenkel, Space-charge limited conduction, Fowler-Nordheim Tunneling, and Schottky Emission. Of all the conduction mechanisms, Schottky emission provided the most reasonable fitting parameters (see below). We note that Fowler-Nordheim tunneling barriers were too small to be physically plausible, and localization parameters from VRH were also unrealistic, and were therefore discarded. As can be seen below in Fig. S3.1, the data is linear in the *log I* vs. $V^{0.5}$ representation[3], as expected for an emission current mechanism. Further, as explained in the manuscript, many points were better described by a two linear segment model, as opposed to a single linear fit model in the applied field range. For model selection, we utilized the Bayesian Information criterion, which is defined as

$$BIC = -2\ln(\hat{L}) + k * \ln(n)$$

where n is the number of datapoints, k is the number of free parameters to be estimated, and $\hat{L}$ is the likelihood function for the model. Lower values of the BIC dictate a more appropriate model choice. In effect, models with large number of free parameters (k) are thus penalized unless their likelihood is substantially greater. Example fits are shown below. Black lines are used where the BIC recommended a single linear function; blue lines are where a two segment linear fit was preferred.

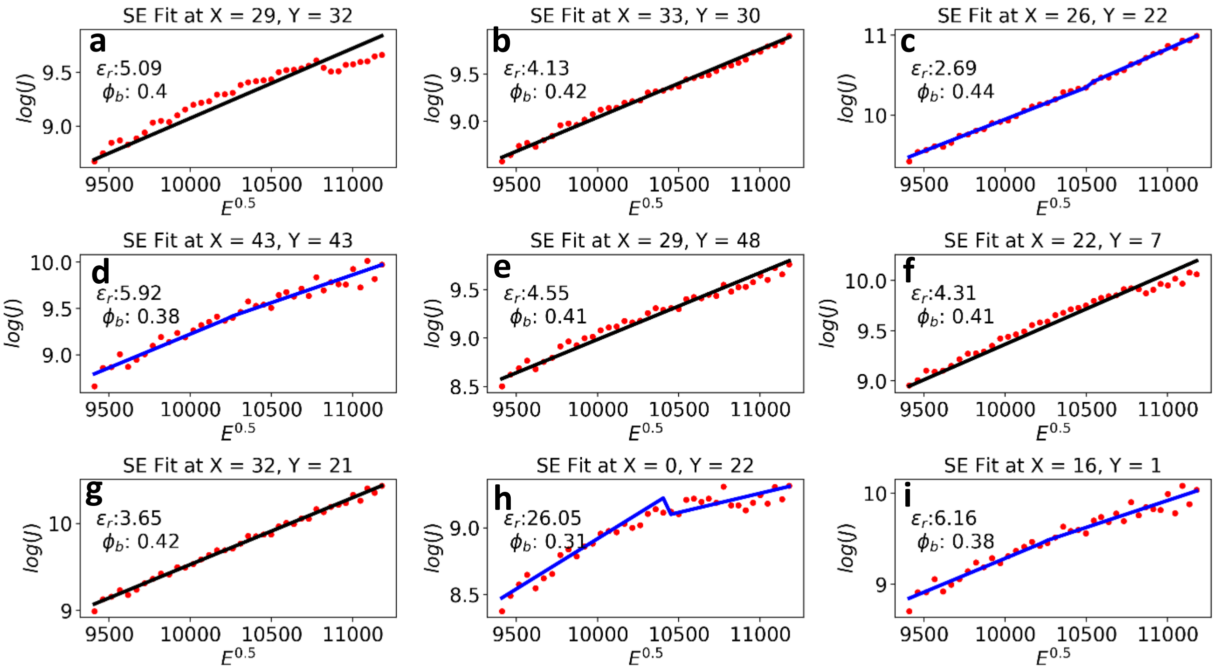

Supplementary Figure 4: Schottky emission fits for 9 randomly selected points out of the 2500 measured. The derived values of the Schottky barrier and the (high-frequency) dielectric constant are shown inset in each plot (a-i). Where the fit is better described by a two segment

linear fit, the dielectric constant and the barrier height have been extracted from the first linear segment.

The fits to other mechanisms (Variable Range Hopping, Schottky-Simmons, Fowler-Nordheim, Space Charge Limited (SCLC) and Poole-Frenkel) are shown below in Fig. Supplementary Figure 5for a representative I-V curve taken at the domain wall (same curve as in Figure 2b in the manuscript). All except two of these mechanisms are unsuitable due to unreasonable values for various calculated parameters (discussed in figure caption). Only SCLC and Schottky emission returned reasonable values for the calculated parameters. We note that regimes of SCLC behavior are typically characterized by slope on a log-log plot of 2 (Mott-Gurney)[4], but higher slopes are possible if one considers a distribution of trap states according to the model of Rose.[5] The SCLC slopes calculated are plotted in Fig. Supplementary Figure 6a. We chose the Schottky-emission instead, because the residuals are slightly less than for the SCLC fits, as seen in the histogram of the residuals in Supplementary Figure 6b.

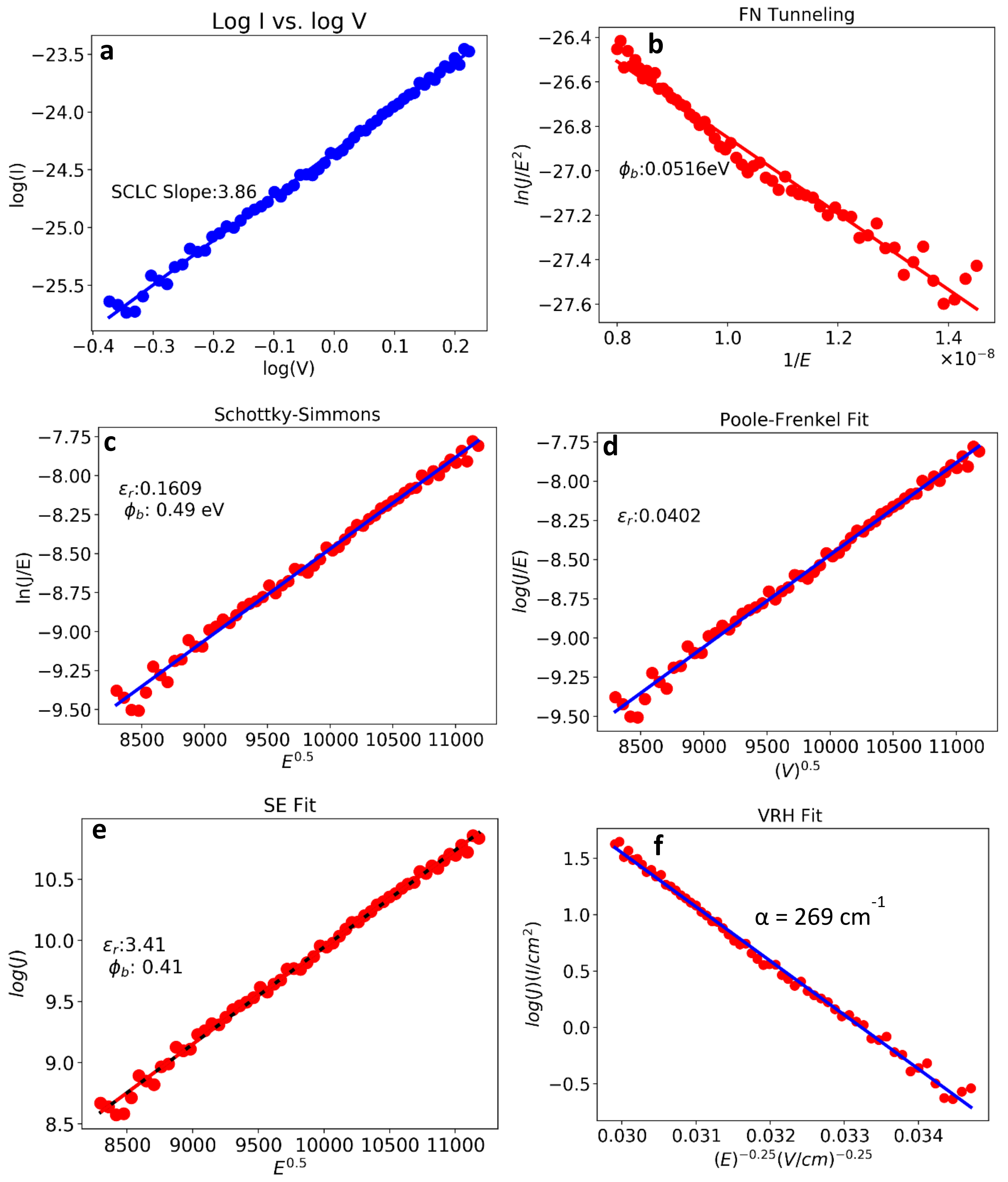

Supplementary Figure 5Fits for the domain wall current to various conduction mechanisms. (a) SCLC representation (log I vs. log V) returns a high value for the slope, although this can be reasonable. (b) Fowler-Nordheim (FN) tunneling (log (J/E^2) vs 1/E) returned unfeasible values for the barrier height. (c) Schottky-Simmons and (d) Poole-Frenkel (both log (J/E) vs √E)) returned unreasonable values for the dielectric constant.(e) Schottky emission

returned better values for the dielectric constant and the barrier height. (f) Variable range hopping, which should be linear in the log(J) vs. $E^{-0.25}$ for high-field[6], appears a good fit. The slope should be proportional to $\frac{81\alpha^4}{16\pi e N_\mu}$ where N_μ is the density of localized states in volume and energy around μ (which we set = 10^{18} cm^{-3} eV^{-1}) to recover α = 269 cm^{-1}, the localization parameter. Since typical localization parameters are on the order of 10^7 cm^{-1}, this value is unfeasible, and thus we excluded VRH as a possible mechanism.

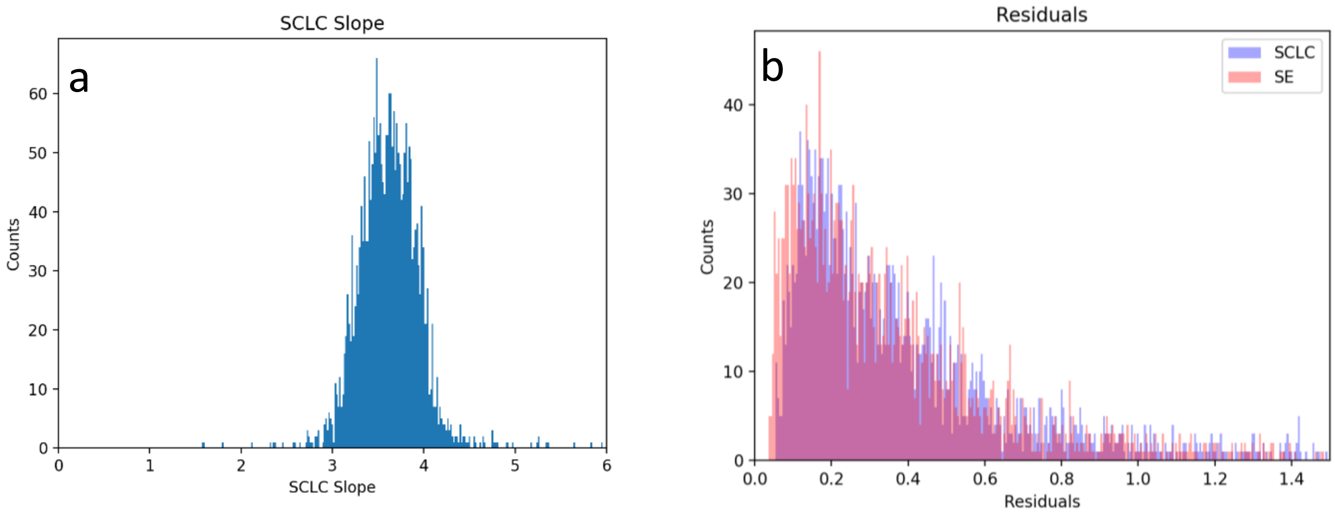

Supplementary Figure 6: SCLC and SE comparison. In (a), we plot the SCLC slopes, which peak between 3-4. For comparison with the fitting with the Schottky emission, we calculated the residuals for every measured I-V curve i.e., calculate the sum of $(\text{data} - \text{fit})^2$, and plot the histogram of these residuals (b). It is observed that the Schottky emission (SE) has slightly lower values for the residuals than the SCLC mechanism, although the difference is small.

Finally, we turn to the evaluation of the fits of the Schottky emission vs. SCLC mechanism. As Fig. S3.3 shows, the values for the slopes of SCLC are almost uniformly greater than 2; however, such a situation is possible if one assumes a distribution of trap states. Nonetheless overall the Schottky emission fit has lower residuals than the SCLC fit, so it was utilized. We would like to emphasize here the purpose of this manuscript is not to claim a particular bulk or interface limited conduction mechanism, which is likely to be heavily defect-dependent (and thus, vary from film to film and under different tip-surface contact conditions), but rather to posit a more general conduction mechanism based on the enhanced field arising from the formation of a polarization nucleus and its interaction with the pre-existing domain walls in the sample. This would be applicable regardless of the specifics of the conduction mechanism chosen above.

Supplementary Note 4 – Phase-Field Modeling

In the phase-field modeling of ferroelectric $BiFeO_3$ thin film, we choose polarization vector $P_i = (P_1, P_2, P_3)$ as the order parameter to describe the ferroelectric domain structures. The domain evolution is modeled by the temporal evolution of P_i obtained by minimizing the total free energy of the system with respect to P_i, [7]

$$\frac{\partial P_i(\boldsymbol{r},t)}{\partial t} = -L\frac{\delta F}{\delta P_i(\boldsymbol{r},t)}, (i = 1 \sim 3) \quad (1)$$

in which t is the time step and L is the kinetic coefficient related to domain wall mobility. The total free energy F of $BiFeO_3$ single crystal is expressed as,

$$F = \int_V [f_{\text{lan}}(P_i) + f_{\text{grad}}(P_{i,j}) + f_{\text{elas}}(P_i, \varepsilon_{ij}) + f_{\text{elec}}(P_i, E_i)]dV \quad (2)$$

where V is the system volume, ε_{ij} and E_i denote the components of strain and electric field, $P_{i,j} = \partial P_i / x_j$, and $f_{\text{lan}}(P_i)$, $f_{\text{grad}}(P_{i,j})$, $f_{\text{elas}}(P_i, \varepsilon_{ij})$ and $f_{\text{elec}}(P_i, E_i)$ represent the Landau-Devonshire free energy density, the gradient energy density, and elastic energy density and the electrostatic energy density, respectively. Detailed expressions of each of the local free energy densities can be found in literature.[8]

We start with an 10 nm thick rhombohedral $BiFeO_3$ thin film consisting of two preset domain variants R_1+ (111) and R_4+(1-11) separated by 71° domain walls. The film is stress-free on top surface and constrained by the substrate. The tip bias on the film surface is modeled by specifying the electric potential distribution using a Lorentz function,

$$\begin{aligned} \phi_1(x,y) &= 0 \\ \phi_2(x,y) &= \phi_0 \frac{\gamma^2}{(x-x_0)^2 + (y-y_0)^2 + \gamma^2} \end{aligned} \quad (3)$$

where ϕ_1 and ϕ_2 represent the electric potential distribution on bottom and top surface respectively, ϕ_0 is the tip bias, (x_0, y_0) is the tip position and γ is the half width half magnitude (HWHM) of the tip. Supplementary Equation 1) is solved by a semi-implicit spectral method[9] combined with Chebyshev collocation method[10] based on a 3D mesh size of $128\Delta x \times 128\Delta y \times 20\Delta z$. Periodic boundary conditions are applied along x and y with $\Delta x = \Delta y$ =1.0nm. Along z direction the grid size (Δz) is non-uniform and a general boundary is applied. The temperature is T=25°C. We assumed an isotropic dielectric constant to be 50. The parameters and energy coefficients of $BiFeO_3$ used in the simulation are listed in Supplementary Table 1.

Supplementary Table 1, Parameters and energy coefficients of $BiFeO_3$ used in the phase-field simulation

**Landau coefficients**		**Elastic coefficients**		**Parameters in the simulation**	
$\alpha_1 (10^8 C^{-2} m^2 N)$	-3.738	c_{11}(GPa)	228	T (°C)	25
$\alpha_{11} (10^8 C^{-4} m^6 N)$	2.290	c_{12}(GPa)	128	System size	128×128×20
$\alpha_{12} (10^8 C^{-4} m^6 N)$	3.064	c_{44}(GPa)	65	Film (nm)	10
$\alpha_{111} (10^7 C^{-6} m^{10} N)$	5.992	**Electrostrictive coefficients**		substrate strain	0.0
$\alpha_{112} (10^5 C^{-6} m^{10} N)$	-3.340	$Q_{11} (10^{-2} C^{-2} m^4)$	3.2	ε_r	50
$\alpha_{123} (10^8 C^{-6} m^{10} N)$	-1.778	$Q_{12} (10^{-2} C^{-2} m^4)$	-1.6		
$\alpha_{1111} (10^8 C^{-8} m^{14} N)$	3.92	$Q_{44} (10^{-2} C^{-2} m^4)$	2.015		

Supplementary Figure 7 – divergence in Polarization

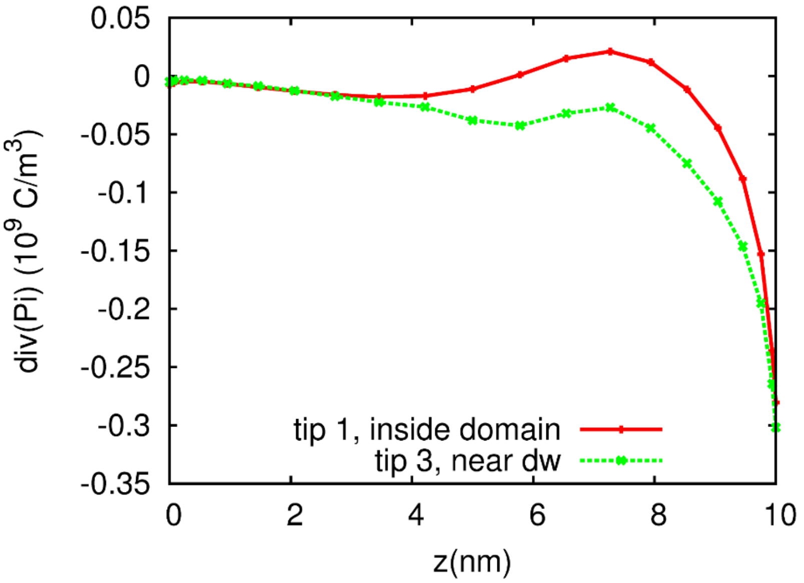

Supplementary Figure 7: Plot of -div(P) along the z-direction directly beneath the tip, when the tip is placed in two different locations as indicated in Figure 4 in the manuscript. Here, the divergence in polarization $(-\mathrm{div}(P_i) = -\left(\frac{dP_x}{dx} + \frac{dP_y}{dy} + \frac{dP_z}{dz}\right))$ is plotted, for the tip in location 1 (within a domain) and the tip in location 3 (next to the domain wall). The plot shows that the divergence is also modified by the twisted reverse-domain structure, but that the modification occurs mostly 2-4 nm into the film. Some change in divergence at the tip apex can also be seen. The divergence is greater for the twisted wall structure (tip 3), slightly at the surface, but more so within the film at a depth of ~2nm.

Supplementary Figure 8 - Asymmetric wall conduction

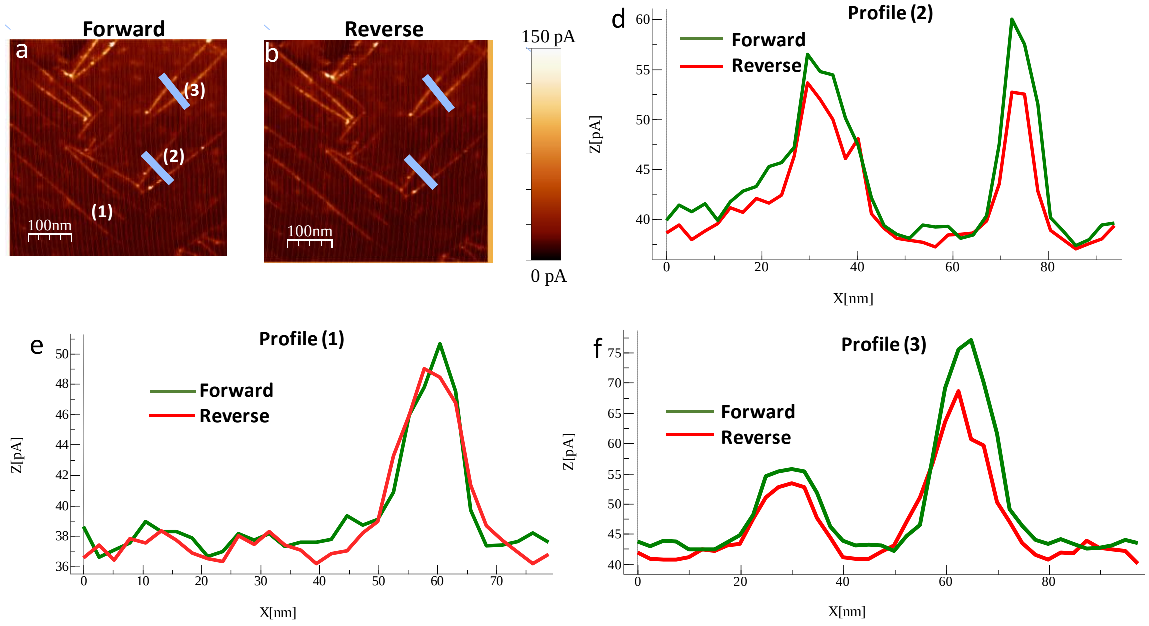

Supplementary Figure 8: Asymmetric conduction at domain walls in an ultra-thin BFO film. **(a)** Forward and **(b)** Reverse traces from c-AFM scans with the sample held at V_{sam} = +1.2V. Line profiles of the lines in (a,b) are shown in **(c,d,e)**. There appears to be a significant asymmetry for the domain wall conduction, as predicted by the phase-field simulations, and forward scan tends to show higher current than the reverse scan direction.

Supplementary Figure 9– Increase in conduction after repeated scanning

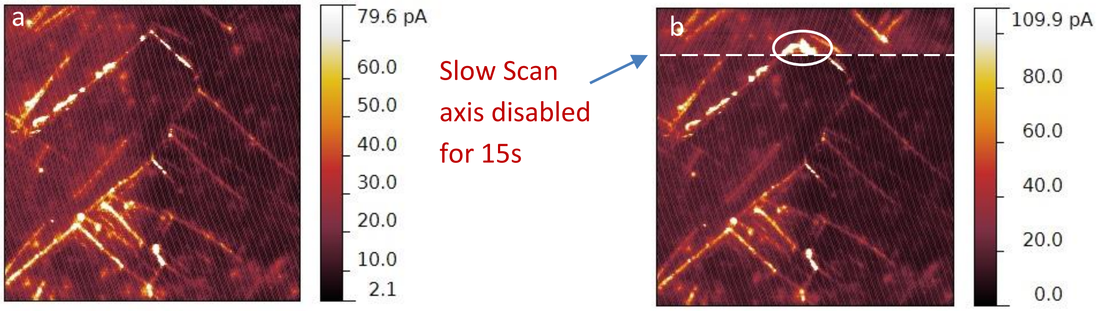

Supplementary Figure 9: Conductivity change by repeated scanning. **(a)** c-AFM with V_{sam} = +1.2V. During the subsequent scan, the slow scan axis was disabled for 15s at the dashed line. The c-AFM scan after this procedure is plotted in **(b)** and clearly shows that the junction in the center of the line (circled) appears to have increased conductivity compared to (a).

Supplementary References

1. Laanait N, Zhang Z, Schlepütz CM, Vila-Comamala J, Highland MJ, Fenter P. Full-field X-ray reflection microscopy of epitaxial thin-films. *Journal of synchrotron radiation* **21**, 1252-1261 (2014).

2. Jesse S, Kalinin SV. Principal component and spatial correlation analysis of spectroscopic-imaging data in scanning probe microscopy. *Nanotechnology* **20**, 085714 (2009).

3. Chiu F-C. A review on conduction mechanisms in dielectric films. *Advances in Materials Science and Engineering* **2014**, 578168 (2014).

4. Morrison F, *et al.* High-field conduction in barium titanate. *Appl Phys Lett* **86**, 152903 (2005).

5. Rose A. Space-charge-limited currents in solids. *Physical Review* **97**, 1538 (1955).

6. van Hapert JJ. Hopping conduction and chemical structure: a study on silicon suboxides. (ed^(eds) (2002).

7. Chen LQ. Phase-field method of phase transitions/domain structures in ferroelectric thin films: A review. *J Amer Cer Soc* **91**, 1835-1844 (2008).

8. Li YL, Hu SY, Liu ZK, Chen LQ. Effect of substrate constraint on the stability and evolution of ferroelectric domain structures in thin films. *Acta Mater* **50**, 395-411 (2002).

9. Chen LQ, Shen J. Applications of semi-implicit Fourier-spectral method to phase field equations. *Comput Phys Commun* **108**, 147-158 (1998).

10. Cao Y, Bhattacharya S, Shen J, Randall C, Chen L. Role of polaron hopping in leakage current behavior of a SrTiO3 single crystal. *J Appl Phys* **114**, 224102 (2013).
